# Supplementary material for: Association between ICU-level variation in arterial blood gas utilization and in-hospital mortality: A retrospective cohort study using the Japanese Intensive care PAtient Database registry
Source: PLoS One. 2026 Jun 9;21(6):e0343186. doi: 10.1371/journal.pone.0343186 (PMC13249154; doi:10.1371/journal.pone.0343186)
Supplement: S1 Table — BMI, Body Mass Index; CVC, Central Venous Catheter; APACHE, Acute Physiology and Chronic Health Evaluation; AKI, Acute Kidney Injury; MV, Mechanical Ventilation; ER, Emergency Room; P/F, PaO₂/ FIO₂. (DOCX) [file pone.0343186.s001.docx]

**S1 Table. Multivariable Linear Regression Model for Predicting Arterial Blood Gas analysis Measurement**

| Variable | Coefficient [95%CI] | P value |
| --- | --- | --- |
| (Intercept) | 3.98 [3.93 to 4.0] | <0.001 |
| Age, years | -0.002 [-0.003 to -0.001] | <0.001 |
| Male | -0.007 [-0.022 to 0.008] | 0.347 |
| BMI, kg/m^2^ | -0.009 [-0.010 to -0.007] | <0.001 |
| CVC | 0.383 [0.365 to 0.401] | <0.001 |
| Heart failure | -0.195 [-0.243 to -0.148] | <0.001 |
| Respiratory failure | 0.061 [0.000 to 0.121] | 0.049 |
| Liver cirrhosis | 0.249 [0.187 to 0.310] | <0.001 |
| Metastatic cancer | 0.033 [-0.010 to 0.077] | 0.134 |
| Immunosuppression | 0.032 [0.002 to 0.063] | 0.038 |
| Maintenance dialysis | -0.052 [-0.083 to -0.020] | 0.001 |
| APACHE III score | 0.002 [0.002 to 0.002] | <0.001 |
| Infection | 0.037 [0.009 to 0.099] | 0.008 |
| Lactate, mmol/L: |  |  |
| 0–<2 (Reference) |  |  |
| 2–<4 | 0.219 [0.202 to 0.236] | <0.001 |
| 4–<6 | 0.285 [0.261 to 0.310] | <0.001 |
| 6–<10 | 0.253 [0.222 to 0.284] | <0.001 |
| ≥10 | 0.193 [0.149 to 0.237] | <0.001 |
| Not measured | 0.306 [0.251 to 0.361] | <0.001 |
| AKI within 24 hours | -0.176 [-0.217 to -0.135] | <0.001 |
| MV within 24 hours | 0.359 [0.341 to 0.378] | <0.001 |
| Admission source: |  |  |
| After elective surgery (Reference) |  |  |
| After emergency surgery | -0.110 [-0.134 to -0.086] | <0.001 |
| Transfer from ER | -0.084 [-0.105 to -0.062] | <0.001 |
| Transfer from Ward | -0.013 [-0.043 to 0.016] | 0.371 |
| Direct from another hospital | -0.334 [-0.378 to -0.289] | <0.001 |
| Others | -0.117 [-0.163 to -0.071] | <0.001 |
| Hospital-to-ICU interval (day) | 0.000 [-0.001 to 0.000] | 0.019 |
| Emergency call | 0.055 [0.010 to 0.099] | 0.016 |
| Primary diagnosis: |  |  |
| Cardiovascular (Reference) |  |  |
| Gastrointestinal | -0.256 [-0.280 to -0.233] | <0.001 |
| Musculoskeletal | -0.163 [-0.241 to -0.084] | <0.001 |
| Endocrine/metabolic | 0.457 [0.413 to 0.501] | <0.001 |
| Neurological | -0.620 [-0.647 to -0.593] | <0.001 |
| Respiratory | -0.347 [-0.374 to -0.320] | <0.001 |
| Trauma | -0.379 [-0.419 to -0.340] | <0.001 |
| Genitourinary | 0.052 [0.007 to 0.097] | <0.001 |
| Others | -0.039 [-0.078 to 0.001] | 0.056 |
| Lowest pH: |  |  |
| >7.35 to 7.45 (Reference) |  |  |
| < 7.15 | 0.198 [0.145 to 0.250] | 0.084 |
| 7.15 to 7.25 | 0.261 [0.226 to 0.296] | <0.001 |
| >7.25 to 7.35 | 0.185 [0.167 to 0.202] | <0.001 |
| >7.45 | -0.47 [-0.51 to -0.43] | <0.001 |
| Not measured | -2.07 [-2.89 to -1.24] | <0.001 |
| Lowest P/F ratio: |  |  |
| ≥400 (Reference) |  |  |
| 300 to 399 | 0.50 [0.48 to 0.53] | <0.001 |
| 200 to 299 | 0.67 [0.65 to 0.69] | <0.001 |
| 100 to 199 | 0.79 [0.77 to 0.82] | <0.001 |
| <100 | 0.80 [0.76 to 0.84] | <0.001 |
| Not measured | -1.23 [-1.67 to -0.80] | <0.001 |
| Highest PaCO_2_, mmHg: |  |  |
| 35 to 45 (Reference) |  |  |
| <35 | -0.165 [-0.192 to -0.139] | <0.001 |
| >45 to 55 | 0.087 [0.068 to 0.105] | <0.001 |
| >55 to 60 | 0.072 [0.029 to 0.116] | <0.001 |
| >60 | 0.114 [0.073 to 0.156] | <0.001 |
| Not measured | -0.668 [-1.44 to 0.106] | 0.230 |

BMI, Body Mass Index; CVC, Central Venous Catheter; APACHE, Acute Physiology and Chronic Health Evaluation; AKI, Acute Kidney Injury; MV, Mechanical Ventilation; ER, Emergency Room; P/F, PaO₂/ F_I_O₂
