# Supplementary material for: Association between ICU-level variation in arterial blood gas utilization and in-hospital mortality: A retrospective cohort study using the Japanese Intensive care PAtient Database registry
Source: PLoS One. 2026 Jun 9;21(6):e0343186. doi: 10.1371/journal.pone.0343186 (PMC13249154; doi:10.1371/journal.pone.0343186)
Supplement: S2 Table — P/F, PaO₂/ FIO₂. (DOCX) [file pone.0343186.s002.docx]

**S2 Table. Detailed patient characteristics corresponding to Table 1**

|  | Overall | Tertile 1 | Tertile 2 | Tertile 3 | p value |
| --- | --- | --- | --- | --- | --- |
| Lactate, mmol/L (%) |  |  |  |  | <0.001 |
| 0–<2 | 48572 (41.3) | 13831 (42.5) | 14540 (39.2) | 20201 (42.2) |  |
| 2–<4 | 39778 (33.8) | 10597 (32.5) | 13000 (35.1) | 16181 (33.8) |  |
| 4–<6 | 14029 (11.9) | 3466 (10.6) | 4602 (12.4) | 5961 (12.4) |  |
| 6–<10 | 8120 (6.9) | 2036 (6.3) | 2711 (7.3) | 3373 (7.0) |  |
| ≥10 | 4080 (3.5) | 1093 (3.4) | 1398 (3.8) | 1589 (3.3) |  |
| Not measured | 2967 (2.5) | 1549 (4.8) | 815 (2.2) | 603 (1.3) |  |
| Admission source: |  |  |  |  | <0.001 |
| After elective surgery (%) | 47978 (40.8) | 11953 (36.7) | 17095 (46.1) | 18930 (39.5) |  |
| After emergency surgery (%) | 16492 (14.0) | 4821 (14.8) | 4840 (13.1) | 6831 (14.3) |  |
| Transfer from ER (%) | 32671 (27.8) | 9791 (30.1) | 9036 (24.4) | 13844 (28.9) |  |
| Transfer from Ward (%) | 13603 (11.6) | 3853 (11.8) | 4262 (11.5) | 5488 (11.5) |  |
| From another hospital (%) | 3608 (3.1) | 1235 (3.8) | 1007 (2.7) | 1366 (2.9) |  |
| Others (%) | 3194 (2.7) | 919 (2.8) | 826 (2.2) | 1449 (3.0) |  |
| Primary diagnosis: |  |  |  |  | <0.001 |
| Cardiovascular (%) | 52784 (44.9) | 12210 (37.5) | 18325 (49.4) | 22249 (46.4) |  |
| Gastrointestinal (%) | 18662 (15.9) | 5711 (17.5) | 5729 (15.5) | 7222 (15.1) |  |
| Musculoskeletal (%) | 987 (0.8) | 162 (0.5) | 393 (1.1) | 432 (0.9) |  |
| Endocrine/metabolic (%) | 3600 (3.1) | 1007 (3.1) | 932 (2.5) | 1661 (3.5) |  |
| Neurological (%) | 12041 (10.2) | 4541 (13.9) | 3603 (9.7) | 3897 (8.1) |  |
| Respiratory (%) | 15677 (13.3) | 4782 (14.7) | 4330 (11.7) | 6565 (13.7) |  |
| Trauma (%) | 4478 (3.8) | 1676 (5.1) | 1152 (3.1) | 1650 (3.4) |  |
| Genitourinary (%) | 3293 (2.8) | 756 (2.3) | 955 (2.6) | 1582 (3.3) |  |
| Others (%) | 6024 (5.1) | 1727 (5.3) | 1647 (4.4) | 2650 (5.5) |  |
| Lowest pH (%) |  |  |  |  | <0.001 |
| < 7.15 | 3404 (2.9) | 906 (2.8) | 1090 (2.9) | 1408 (2.9) |  |
| 7.15–7.25 | 7565 (6.4) | 1778 (5.5) | 2420 (6.5) | 3367 (7.0) |  |
| >7.25–7.35 | 38577 (32.8) | 9511 (29.2) | 12460 (33.6) | 16606 (34.7) |  |
| >7.35–7.45 | 57475 (48.9) | 16558 (50.8) | 17719 (47.8) | 23198 (48.4) |  |
| >7.45 | 5330 (4.5) | 1958 (6.0) | 1568 (4.2) | 1804 (3.8) |  |
| Not measured | 5195 (4.4) | 1861 (5.7) | 1809 (4.9) | 1525 (3.2) |  |
| Lowest P/F ratio (%) |  |  |  |  | <0.001 |
| ≥400 | 12213 (10.4) | 4371 (13.4) | 3475 (9.4) | 4367 (9.1) |  |
| 300–399 | 28265 (24.0) | 7904 (24.3) | 8470 (22.9) | 11891 (24.8) |  |
| 200–299 | 35306 (30.0) | 9257 (28.4) | 11705 (31.6) | 14344 (29.9) |  |
| 100–199 | 29203 (24.8) | 7417 (22.8) | 9372 (25.3) | 12414 (25.9) |  |
| <100 | 7344 (6.2) | 1754 (5.4) | 2234 (6.0) | 3356 (7.0) |  |
| Not measured | 5215 (4.4) | 1869 (5.7) | 1810 (4.9) | 1536 (3.2) |  |
| Highest PaCO_2_, mmHg (%) |  |  |  |  | <0.001 |
| <35 | 11690 (9.9) | 3773 (11.6) | 3307 (8.9) | 4610 (9.6) |  |
| 35–45 | 60125 (51.2) | 16697 (51.3) | 19247 (51.9) | 24181 (50.5) |  |
| >45–55 | 31536 (26.8) | 7854 (24.1) | 10016 (27.0) | 13666 (28.5) |  |
| >55–60 | 3660 (3.1) | 942 (2.9) | 1082 (2.9) | 1636 (3.4) |  |
| >60 | 5336 (4.5) | 1441 (4.4) | 1605 (4.3) | 2290 (4.8) |  |
| Not measured | 5199 (4.4) | 1865 (5.7) | 1809 (4.9) | 1525 (3.2) |  |

P/F, PaO₂/ F_I_O₂
