# Supplementary material for: Association between ICU-level variation in arterial blood gas utilization and in-hospital mortality: A retrospective cohort study using the Japanese Intensive care PAtient Database registry
Source: PLoS One. 2026 Jun 9;21(6):e0343186. doi: 10.1371/journal.pone.0343186 (PMC13249154; doi:10.1371/journal.pone.0343186)
Supplement: S3 Table — BMI, Body Mass Index; CVC, Central Venous Catheter; APACHE, Acute Physiology and Chronic Health Evaluation; AKI, Acute Kidney Injury; MV, Mechanical Ventilation; ER, Emergency Room; P/F, PaO₂/ FIO₂. (DOCX) [file pone.0343186.s003.docx]

**S3 Table. Full multilevel logistic regression model corresponding to Table 3**

| Variable | Odds ratio [95%CI] | p value |
| --- | --- | --- |
| Tertile 1 (Reference) |  |  |
| Tertile 2 | 0.942 [0.807, 1.100] | 0.448 |
| Tertile 3 | 0.874 [0.751, 1.017] | 0.082 |
| Age, years | 1.005 [1.003, 1.006] | <0.001 |
| Male | 1.047 [1.000, 1.096] | 0.048 |
| BMI, kg/m^2^ | 0.990 [0.985, 0.995] | <0.001 |
| CVC | 1.374 [1.299, 1.453] | <0.001 |
| Heart failure | 1.710 [1.509, 1.938] | <0.001 |
| Respiratory failure | 1.388 [1.215, 1.585] | <0.001 |
| Liver cirrhosis | 1.434 [1.248, 1.648] | <0.001 |
| Metastatic cancer | 1.671 [1.504, 1.857] | <0.001 |
| Immunosuppression | 1.508 [1.396, 1.629] | <0.001 |
| Maintenance dialysis | 1.448 [1.337, 1.568] | <0.001 |
| APACHE III score | 1.034 [1.033, 1.035] | <0.001 |
| Infection | 0.754 [0.704, 0.807] | <0.001 |
| Lactate, mmol/L (%): |  |  |
| 0–<2 (Reference) |  |  |
| 2–<4 | 1.206 [1.141, 1.274] | <0.001 |
| 4–<6 | 1.378 [1.280, 1.484] | <0.001 |
| 6–<10 | 1.750 [1.614, 1.898] | <0.001 |
| ≥10 | 2.523 [2.284, 2.787] | <0.001 |
| Not measured | 1.489 [1.256, 1.766] | <0.001 |
| AKI within 24 hours | 1.452 [1.336, 1.578] | <0.001 |
| MV within 24 hours | 0.850 [0.802, 0.901] | <0.001 |
| Admission source: |  |  |
| After elective surgery (Reference) |  |  |
| After emergency surgery | 3.128 [2.866, 3.414] | <0.001 |
| Transfer from ER | 3.480 [3.198, 3.786] | <0.001 |
| Transfer from Ward | 5.129 [4.685, 5.614] | <0.001 |
| From another hospital | 4.485 [3.947, 5.097] | <0.001 |
| Others | 5.441 [4.823, 6.137] | <0.001 |
| Hospital-to-ICU interval | 1.006 [1.005, 1.008] | <0.001 |
| Emergency call | 0.827 [0.751, 0.910] | <0.001 |
| Primary diagnosis: |  |  |
| Cardiovascular (Reference) |  |  |
| Gastrointestinal | 1.153 [1.067, 1.245] | <0.001 |
| Musculoskeletal | 1.697 [1.269, 2.267] | <0.001 |
| Endocrine/metabolic | 0.398 [0.344, 0.460] | <0.001 |
| Neurological | 1.400 [1.287, 1.522] | <0.001 |
| Respiratory | 1.856 [1.714, 2.011] | <0.001 |
| Trauma | 1.222 [1.082, 1.380] | 0.001 |
| Genitourinary | 0.728 [0.611, 0.867] | <0.001 |
| Others | 1.230 [1.113, 1.359] | <0.001 |
| Lowest pH (%): |  |  |
| <7.35–7.45 (Reference) |  |  |
| < 7.15 | 0.962 [0.857, 1.079] | 0.507 |
| 7.15–7.25 | 1.079 [0.992, 1.174] | 0.076 |
| >7.25–7.35 | 0.995 [0.940, 1.052] | 0.850 |
| >7.45 | 1.231 [1.122, 1.350] | <0.001 |
| Not measured | 0.204 [0.019, 2.194] | 0.190 |
| Lowest P/F ratio (%): |  |  |
| ≥400 (Reference) |  |  |
| 300–399 | 0.892 [0.811, 0.980] | 0.018 |
| 200–299 | 1.004 [0.917, 1.100] | 0.929 |
| 100–199 | 1.040 [0.947, 1.142] | 0.415 |
| <100 | 1.397 [1.252, 1.559] | <0.001 |
| Not measured | 1.786 [0.624, 5.106] | 0.280 |
| Highest PaCO_2_, mmHg (%): |  |  |
| 35–45 (Reference) |  |  |
| <35 | 1.243 [1.162, 1.329] | <0.001 |
| >45–55 | 1.055 [0.995, 1.120] | 0.073 |
| >55–60 | 1.239 [1.109, 1.385] | <0.001 |
| >60 | 1.398 [1.269, 1.540] | <0.001 |
| Not measured | 4.139 [0.505, 33.935] | 0.186 |
| Number of hospital beds [IQR] | 1.000 [0.999, 1.000] | 0.192 |
| Number of ICU beds [IQR] | 0.992 [0.983, 1.001] | 0.089 |
| Academic hospital (%) | 1.098 [0.965, 1.249] | 0.155 |
| Intensivist-to-ICU bed ratio [IQR] | 0.950 [0.854, 1.056] | 0.338 |
| ICU nurse-to-ICU bed ratio [IQR] | 0.981 [0.950, 1.014] | 0.265 |

BMI, Body Mass Index; CVC, Central Venous Catheter; APACHE, Acute Physiology and Chronic Health Evaluation; AKI, Acute Kidney Injury; MV, Mechanical Ventilation; ER, Emergency Room; P/F, PaO₂/ F_I_O₂
