# Supplementary material for: Association between ICU-level variation in arterial blood gas utilization and in-hospital mortality: A retrospective cohort study using the Japanese Intensive care PAtient Database registry
Source: PLoS One. 2026 Jun 9;21(6):e0343186. doi: 10.1371/journal.pone.0343186 (PMC13249154; doi:10.1371/journal.pone.0343186)
Supplement: S1 File — (PDF) [file pone.0343186.s004.pdf]

```
### data ###----
install.packages("readr")
library(readr)
# patient information N=324473
df_patient <- read_csv("request_data_patient_29.csv",
                                    locale = locale(encoding = "Shift-JIS"))
#institution information
df_institution_15 <- read_csv(
  "request_data_institution_2015.csv",
  locale = locale(encoding = "Shift-JIS")
)
df_institution_16 <- read_csv(
  "request_data_institution_2016.csv",
  locale = locale(encoding = "Shift-JIS")
)
df_institution_17 <- read_csv(
  "request_data_institution_2017.csv",
  locale = locale(encoding = "Shift-JIS")
)
df_institution_18 <- read_csv(
  "request_data_institution_2018.csv",
  locale = locale(encoding = "Shift-JIS")
)
df_institution_19 <- read_csv(
  "request_data_institution_2019.csv",
  locale = locale(encoding = "Shift-JIS")
)
df_institution_20 <- read_csv(
  "request_data_institution_2020.csv",
  locale = locale(encoding = "UTF-8")
)
df_institution_21 <- read_csv(
  "request_data_institution_2021.csv",
  locale = locale(encoding = "Shift-JIS")
)
df_institution_22 <- read_csv(
```

```
  "request_data_institution_2022.csv",
  locale = locale(encoding = "Shift-JIS")
)
### data cleaning   ###----

# tidyverse
install.packages("tidyverse")
library(tidyverse)

## marge（patient and institution）
#+year
df_institution_15 %>% mutate("年度" = "2015") -> df_institution_15
df_institution_16 %>% mutate("年度" = "2016") -> df_institution_16
df_institution_17 %>% mutate("年度" = "2017") -> df_institution_17
df_institution_18 %>% mutate("年度" = "2018") -> df_institution_18
df_institution_19 %>% mutate("年度" = "2019") -> df_institution_19
df_institution_20 %>% mutate("年度" = "2020") -> df_institution_20
df_institution_21 %>% mutate("年度" = "2021") -> df_institution_21
df_institution_22 %>% mutate("年度" = "2022") -> df_institution_22

# character
df_patient <- df_patient %>%
  mutate(年度　= as.character(年度))



#marge
merged_data_15 <- merge(df_patient, df_institution_15, by= c("施設固有 ID" , "年度"))
merged_data_16 <- merge(df_patient, df_institution_16, by= c("施設固有 ID" , "年度"))
merged_data_17 <- merge(df_patient, df_institution_17, by= c("施設固有 ID" , "年度"))
merged_data_18 <- merge(df_patient, df_institution_18, by= c("施設固有 ID" , "年度"))
merged_data_19 <- merge(df_patient, df_institution_19, by= c("施設固有 ID" , "年度"))
merged_data_20 <- merge(df_patient, df_institution_20, by= c("施設固有 ID" , "年度"))
merged_data_21 <- merge(df_patient, df_institution_21, by= c("施設固有 ID" , "年度"))
merged_data_22 <- merge(df_patient, df_institution_22, by= c("施設固有 ID" , "年度"))

#delete
```

```
merged_data_22_formerge <- merged_data_22 %>% select(-"主な形態", -"運用体制")

#marge N=32439
merged_data_all <- rbind(merged_data_15, merged_data_16, merged_data_17,
merged_data_18,
                         merged_data_19, merged_data_20, merged_data_21,
merged_data_22_formerge)

# character
merged_data_all <- merged_data_all %>%
  mutate(年度 = as.character(年度))




# df cleaning
#columns_to_remove
columns_to_remove <- c( "施設番号.x", "副病名コード 1",
                        "副病名コード 2", "副病名コード 3", "副病名コード 4",
                        "副病名コード 5", "人工呼吸開始 2 日", "人工呼吸開始 2 時刻",
                        "人工呼吸終了 2 日", "人工呼吸終了 2 時刻",
                        "人工呼吸開始 3 日", "人工呼吸開始 3 時刻",
                        "人工呼吸終了 3 日", "人工呼吸終了 3 時刻",
                        "人工呼吸開始 4 日", "人工呼吸開始 4 時刻",
                        "人工呼吸終了 4 日", "人工呼吸終了 4 時刻",
                        "人工呼吸開始 5 日", "人工呼吸開始 5 時刻",
                        "人工呼吸終了 5 日", "人工呼吸終了 5 時刻",
                        "FiO2_小児", "PaO2_小児", "人工呼吸_小児", "術後の回復目的",
                        "高リスク診断名", "低リスク診断名", "年齢詳細_小児",
                        "施設番号.y", "収縮期血圧", "瞳孔散大", "BE", "人工心肺使用後",
                        "PIM2 スコア", "PIM2 院内予測死亡率", "超高リスク診断名",
                        "心臓手術もしくは心カテ術後", "小児主病名コード", "小児副病
名コード 1",
                        "小児副病名コード 2", "小児副病名コード 3", "小児副病名コー
ド 4",
                        "小児副病名コード 5", "PF 比", "PIM3 スコア", "PIM3 院内予測
死亡率",
```

```
                                          "pSOFA スコア", "専任・専従医師数_平日_午前 10 時", "専任・
専従医師数_平日_午後 8 時",
                                          "専任・専従医師数_平日_午前 4 時", "専任・専従医師数_休日_
午前 10 時",
                                          "専任・専従医師数_休日_午後 8 時", "専任・専従医師数_休日_
午前 4 時",
                                          "専任・専従以外の医師数_平日_午前 10 時", "専任・専従以外の
医師数_平日_午後 8 時",
                                          "専任・専従以外の医師数_平日_午前 4 時", "専任・専従以外の
医師数_休日_午前 10 時",
                                          "専任・専従以外の医師数_休日_午後 8 時", "専任・専従以外の
医師数_休日_午前 4 時",
                                          "看護師数_平日_午前 10 時", "看護師数_平日_午後 8 時", "看護
師数_平日_午前 4 時",
                                          "看護師数_休日_午前 10 時", "看護師数_休日_午後 8 時", "看護
師数_休日_午前 4 時")

#delete (colums_to_remove)
merged_data_all <- merged_data_all[, -which(names(merged_data_all) %in%
columns_to_remove)]

# colnames
colnames(merged_data_all)

# rename
merged_data_all <- merged_data_all %>%

   rename( "id" = "施設固有 ID", "year" = "年度", "age" = "年齢", "sex" = "性別",
              "adm_day" = "入院日", "disc_day" = "退院日", "hosp_outcome" = "退院時転帰",
              "weight" = "体重", "height" = "身長", "icu_adm_day" = "入室日",
              "icu_adm_time" = "入室日時", "type_adm" = "入室形式", "route_adm" = "入室経
路",
              "categ_adm" = "入室区分", "em_call" = "緊急コール", "post_pcr" = "心停止蘇生
後",
              "re_adm" = "再入室", "disease_code" = "主病名コード", "icu_disc_day" = "退室
日",
```

"icu_disc_time" = "退室日時" , "icu_outcome" = "退室時転帰",
"a_line" = "動脈圧ライン", "cv_line" = "中心静脈ライン",
"mec_vent_start_day" = "人工呼吸開始 1 日", "mec_vent_start_time" = "人工呼吸開始 1 時刻",
"mec_vent_finish_day" = "人工呼吸終了 1 日", "mec_vent_finish_time" = "人工呼吸終了 1 時刻",
"nppv" = "NPPV", "tracheotomy" = "気管切開", "tracheotomy_day" = "気管切開施行日",
"iabp" = "IABP", "va_ecmo" = "PCPS", "vv_ecmo" = "VV_ECMO", "irrt" = "間欠腎代替療法",
"crrt" = "持続腎代替療法", "pex" = "血漿交換", "pmx" = "PMX", "other_purification" = "その他の血液浄化",
"aids" = "AIDS", "heart_failure" = "心不全", "resp_failure" = "呼吸不全",
"liver_failure" = "肝不全", "liver_cirrhosis" = "肝硬変", "aml" = "AML_MM",
"lymphoma" = "リンパ腫" , "meta" = "癌転移", "immunosup" = "免疫抑制",
"dialysis" = "維持透析", "pr_max" = "脈拍ー最高", "pr_min" = "脈拍ー最低",
"sbp_max" = "収縮期血圧ー最高", "sbp_min" = "収縮期血圧ー最低",
"mbp_max" = "平均血圧ー最高", "mbp_min" = "平均血圧ー最低",
"dbp_max" = "拡張期血圧ー最高", "dbp_min" = "拡張期血圧ー最低",
"bt_max" = "体温ー最高", "bt_min" = "体温ー最低",
"rr_max" = "呼吸数ー最高", "rr_min" = "呼吸数ー最低", "gcs_e" = "GCS_E",
"gcs_v" = "GCS_V", "gcs_m" = "GCS_M", "ht_max" = "ヘマトクリットー最高",
"ht_min" = "ヘマトクリットー最低",　"wbc_max" = "白血球数ー最高",
"wbc_min" = "白血球数ー最低", "cre_max" = "クレアチニンー最高",
"cre_min" = "クレアチニンー最低", "bun_max" = "BUNー最高", "urin_vol" = "入室後 24 時間の尿量",
"na_max" = "Naー最高", "na_min" = "Naー最低", "k_max" = "Kー最高",
"k_min" = "Kー最低", "alb_max" = "アルブミンー最高", "alb_min" = "アルブミンー最低",
"bil_max" = "ビリルビンー最高", "bs_max" = "血糖ー最高", "bs_min" = "血糖ー最低",
"aki" = "入室後 24 時間以内の AKI", "mec_vent_24h" = "入室後 24 時間以内の人工呼吸",
"pac" = "入室後 24 時間以内の PAC", "re_cabg" = "CABG 再手術", "num_graft" = "グラフト本数",
"fio2_1" = "FiO2_1", "pao2_1" = "PaO2_1", "paco2_1" = "PaCO2_1", "ph_1" =

"pH_1",
"fio2_2" = "FiO2_2", "pao2_2" = "PaO2_2", "paco2_2" = "PaCO2_2", "ph_2" = "pH_2",
"fio2_3" = "FiO2_3", "pao2_3" = "PaO2_3", "paco2_3" = "PaCO2_3", "ph_3" = "pH_3",
"fio2_4" = "FiO2_4", "pao2_4" = "PaO2_4", "paco2_4" = "PaCO2_4", "ph_4" = "pH_4",
"fio2_5" = "FiO2_5", "pao2_5" = "PaO2_5", "paco2_5" = "PaCO2_5", "ph_5" = "pH_5",
"fio2_6" = "FiO2_6", "pao2_6" = "PaO2_6", "paco2_6" = "PaCO2_6", "ph_6" = "pH_6",
"apache2" = "APACHEII スコア", "apache2_mortality" = "APACHEII 院内予測死亡率",
"apache3" = "APACHEIII スコア", "apache3_mortality" = "APACHEIII 院内予測死亡率",
"saps" = "SAPSII スコア", "saps_mortality" = "SAPSII 院内予測死亡率",
"tracheostomized" = "入室時気管切開", "hfnc" = "HFNC", "plt_min" = "血小板ー最低",
"dob" = "DOB", "doa" = "DOA", "nad" = "NOR", "ad" = "ADR", "lac" = "乳酸値",
"sofa" = "SOFA スコア", "jrod_mortality" = "JROD 予測死亡率",
"adm_hash" = "入院 hash", "patient_hash" = "患者 hash",
"num_bed_hosp" = "許可病床数", "hosp_type" = "病院のタイプ",
"num_ecmo" = "経皮的心肺補助装置（膜型人工肺を含む）の保有台数",
"num_crrt" = "持続血液ろ過透析装置の保有台数", "num_bed_icu" = "ICU 加算認可病床数",
"area_bed" = "ICU ベッドの平均床面積", "area_open" = "一床あたりの面積（オープンベッド）",
"area_closed" = "一床当たりの面積（個室）",
"num_fulltime_specialist" = "専従医かつ集中治療医学会認定専門医数",
"num_fulltime_unspecialist" = "専門医以外の専従医数",
"num_fulltime_over5year" = "ICU 勤務 5 年以上の専従医数",
"num_specialist" = "集中治療医学会認定専門医数（非専従含む院内総数）",
"num_rotater" = "他部署からのローテーション医師数（月平均）",
"num_resident" = "研修医数（月平均）", "num_fulltime_nurse" = "看護師_専従人数",

"num_nurse_certified_cc" = "集中ケア認定看護師数",
"num_nurse_certified_em" = "救急看護認定看護師数",
"num_nurse_specialized" = "急性・重症患者看護専門看護師数",
"num_me_hosp" = "臨床工学技士_人数（病院全体）", "num_me_24h"= "臨床工学技士_24 時間常駐の有無",
"num_fulltime_me" = "臨床工学技士_ICU 専任数",
"time_me" = "臨床工学技士_1 週間のうちで ICU で一人以上が業務している総時間数",
"num_fulltime_pharm" ="薬剤師_ICU 専任数",
"time_pharm" = "薬剤師_1 週間のうちで ICU で一人以上が業務している総時間数"
)

```
## clumn cleaning
#
unique_values_sex <- unique(merged_data_all$sex)
unique_values_hosp_outcome <- unique(merged_data_all$hosp_outcome)
unique_values_type_adm <- unique(merged_data_all$type_adm)
unique_values_route_adm <- unique(merged_data_all$route_adm)
unique_values_categ_adm <- unique(merged_data_all$categ_adm)
unique_values_em_call <- unique(merged_data_all$em_call)
unique_values_post_pcr <- unique(merged_data_all$post_pcr)
unique_values_re_adm <- unique(merged_data_all$re_adm)
unique_values_icu_outcome <- unique(merged_data_all$icu_outcome)
unique_values_a_line <- unique(merged_data_all$a_line)
unique_values_cv_line <- unique(merged_data_all$cv_line)
unique_values_nppv <- unique(merged_data_all$nppv)
unique_values_tracheotomy <- unique(merged_data_all$tracheotomy)
unique_values_iabp <- unique(merged_data_all$iabp)
unique_values_va_ecmo <- unique(merged_data_all$va_ecmo)
unique_values_vv_ecmo <- unique(merged_data_all$vv_ecmo)
unique_values_irrt <- unique(merged_data_all$irrt)
unique_values_crrt <- unique(merged_data_all$crrt)
```

```
unique_values_pex <- unique(merged_data_all$pex)
unique_values_pmx <- unique(merged_data_all$pmx)
unique_values_aids <- unique(merged_data_all$aids)
unique_values_heart_failure <- unique(merged_data_all$heart_failure)
unique_values_resp_failure <- unique(merged_data_all$resp_failure)
unique_values_liver_failure <- unique(merged_data_all$liver_failure)
unique_values_liver_cirrhosis <- unique(merged_data_all$liver_cirrhosis)
unique_values_aml <- unique(merged_data_all$aml)
unique_values_lymphoma <- unique(merged_data_all$lymphoma)
unique_values_meta <- unique(merged_data_all$meta)
unique_values_immunosup <- unique(merged_data_all$immunosup)
unique_values_dialysis <- unique(merged_data_all$dialysis)
unique_values_tracheostomized <- unique(merged_data_all$tracheostomized)
unique_values_hfnc <- unique(merged_data_all$hfnc)
unique_values_dob <- unique(merged_data_all$dob)
unique_values_doa <- unique(merged_data_all$doa)
unique_values_nad <- unique(merged_data_all$nad)
unique_values_ad <- unique(merged_data_all$ad)
unique_values_mec_vent_24h <- unique(merged_data_all$mec_vent_24h)
unique_values_hosp_type <- unique(merged_data_all$hosp_type)
unique_values_num_me_24h <- unique(merged_data_all$num_me_24h)

# list
results <- list(
  sex = unique_values_sex,
  hosp_outcome =   unique_values_hosp_outcome,
  type_adm = unique_values_type_adm,
  route_adm = unique_values_route_adm,
  categ_adm = unique_values_categ_adm,
  em_call = unique_values_em_call,
  post_pcr = unique_values_post_pcr,
  re_adm = unique_values_re_adm,
  icu_outcome = unique_values_icu_outcome,
  a_line = unique_values_a_line,
  cv_line = unique_values_cv_line,
  nppv = unique_values_nppv,
```

```
    tracheotomy = unique_values_tracheotomy,
    iabp = unique_values_iabp,
    va_ecmo = unique_values_va_ecmo,
    vv_ecmo = unique_values_vv_ecmo,
    irrt = unique_values_irrt,
    crrt = unique_values_crrt,
    pex = unique_values_pex,
    pmx = unique_values_pmx,
    aids = unique_values_aids,
    heart_failure = unique_values_heart_failure,
    resp_failure = unique_values_resp_failure,
    liver_failure = unique_values_liver_failure,
    liver_cirrhosis = unique_values_liver_cirrhosis,
    aml = unique_values_aml,
    lymphoma = unique_values_lymphoma,
    meta = unique_values_meta,
    immunosup = unique_values_immunosup,
    dialysis = unique_values_dialysis,
    tracheostomized = unique_values_tracheostomized,
    hfnc= unique_values_hfnc,
    dob = unique_values_dob,
    doa = unique_values_doa,
    nad = unique_values_nad,
    ad   = unique_values_ad,
    mec_vent_24h = unique_values_mec_vent_24h,
    hosp_type = unique_values_hosp_type,
    num_me_24h = unique_values_num_me_24h
)

print(results)

## 0/1
# sex
merged_data_all$sex <- ifelse(merged_data_all$sex == "男性", 1,

                              ifelse(merged_data_all$sex == "女性", 0, NA))
```

```
# hosp_outcome
merged_data_all$hosp_outcome <- ifelse(merged_data_all$hosp_outcome == "転院", 2,
                                       ifelse(merged_data_all$hosp_outcome == "死
亡", 1,
                                              ifelse(merged_data_all$hosp_outcome
== "生存", 0, NA)))

#
merged_data_all <- merged_data_all %>%
  mutate(hosp_outcome = ifelse(hosp_outcome == 2, 0, hosp_outcome))

#
unique(merged_data_all$hosp_outcome)


# type_adm
merged_data_all$type_adm <- ifelse(merged_data_all$type_adm == "ICU での手技", 2,
                                   ifelse(merged_data_all$type_adm == "緊急", 1,
                                          ifelse(merged_data_all$type_adm == "予定
", 0, NA)))


# route_adm
merged_data_all$route_adm <-  ifelse(merged_data_all$route_adm == "PICU", 5,
                                     ifelse(merged_data_all$route_adm == "HCU", 5,
                                            ifelse(merged_data_all$route_adm == "他
の ICU", 5,

ifelse(merged_data_all$route_adm == "NICU", 5,

ifelse(merged_data_all$route_adm == "CCU", 5,

ifelse(merged_data_all$route_adm == "転院直入", 4,

ifelse(merged_data_all$route_adm == "病棟", 3,
```

```
ifelse(merged_data_all$route_adm == "救急外来", 2,

ifelse(merged_data_all$route_adm == "手術室", 1, NA))))))))

# categ_adm
merged_data_all$categ_adm <- ifelse(merged_data_all$categ_adm == "予定手術", 2,
ifelse(merged_data_all$categ_adm == "緊急手術", 1, ifelse(merged_data_all$categ_adm ==
"非手術", 0, NA)))



# make new_type_adm
merged_data_all <- merged_data_all %>%
  mutate(
    new_type_adm = ifelse(route_adm == 1 & categ_adm == 2, 0,   # route_adm が 1 で
categ_adm が 2 のとき new_は 0
                          ifelse(route_adm == 1 & categ_adm == 1, 1,   # route_adm が
1 で categ_acm が 1 のとき new_は 1
                                 route_adm)))                    # それ以外の場
合は new_には route_adm の値をそのまま




# em_call
merged_data_all$em_call <-   ifelse(merged_data_all$em_call == "コードブルー", 1,
                                    ifelse(merged_data_all$em_call == "RRT/MET", 1,
                                           ifelse(merged_data_all$em_call == "No" |
merged_data_all$em_call == "no", 0, NA)))



# post_pcr
merged_data_all$post_pcr <- ifelse(merged_data_all$post_pcr == "Yes", 1,
                                    ifelse(merged_data_all$post_pcr == "No", 0, NA))

# re_adm
merged_data_all$re_adm <- ifelse(merged_data_all$re_adm == "Yes", 1,
                                  ifelse(merged_data_all$re_adm == "No", 0, NA))
```

```
# icu_outcome　死亡が 0 なので注意
merged_data_all$icu_outcome <-　ifelse(merged_data_all$icu_outcome == "PICU", 8,
                                                          ifelse(merged_data_all$icu_outcome ==
"HCU", 7,
                                                                   ifelse(merged_data_all$icu_outcome
== "他の ICU", 6,

ifelse(merged_data_all$icu_outcome == "NICU", 5,

ifelse(merged_data_all$icu_outcome == "CCU", 4,

ifelse(merged_data_all$icu_outcome == "転院", 3,

ifelse(merged_data_all$icu_outcome == "退院", 2,

ifelse(merged_data_all$icu_outcome == "病棟", 1,

ifelse(merged_data_all$icu_outcome == "死亡", 0, NA))))))))




# a_line
merged_data_all$a_line <- ifelse(merged_data_all$a_line == "Yes", 1,
                                              ifelse(merged_data_all$a_line == "No", 0, NA))
# cv_line
merged_data_all$cv_line <- ifelse(merged_data_all$cv_line == "Yes", 1,
                                               ifelse(merged_data_all$cv_line == "No", 0, NA))

# nppv
merged_data_all$nppv <- ifelse(merged_data_all$nppv == "Yes", 1,
                                            ifelse(merged_data_all$nppv == "No", 0, NA))
# tracheotomy
merged_data_all$tracheotomy   <- ifelse(merged_data_all$tracheotomy   == "外科的", 2,
                                                           ifelse(merged_data_all$tracheotomy   == "
```

```
経皮的", 1,
                                              ifelse(merged_data_all$tracheotomy
== "No", 0, NA)))

# tracheotomy
merged_data_all <- merged_data_all %>%
  mutate(tracheotomy = ifelse(tracheotomy == 2, 1, tracheotomy))

# iabp
merged_data_all$iabp <- ifelse(merged_data_all$iabp == "Yes", 1,
                               ifelse(merged_data_all$iabp == "No", 0, NA))

# va_ecmo
merged_data_all$va_ecmo <- ifelse(merged_data_all$va_ecmo == "Yes", 1,
                                  ifelse(merged_data_all$va_ecmo == "No", 0, NA))

# vv_ecmo
merged_data_all$vv_ecmo <- ifelse(merged_data_all$vv_ecmo == "Yes", 1,
                                  ifelse(merged_data_all$vv_ecmo == "No", 0, NA))

# ecmo
merged_data_all <- merged_data_all %>%
  mutate(ecmo = va_ecmo + vv_ecmo)

merged_data_all$ecmo <- ifelse(merged_data_all$ecmo == "2", 1,
                               ifelse(merged_data_all$ecmo == "1", 1,
                                      ifelse(merged_data_all$ecmo == "0", 0, NA)))



# irrt
merged_data_all$irrt <- ifelse(merged_data_all$irrt == "Yes", 1,
                               ifelse(merged_data_all$irrt == "No", 0, NA))

# crrt
merged_data_all$crrt <- ifelse(merged_data_all$crrt == "Yes", 1,
                               ifelse(merged_data_all$crrt == "No", 0, NA))
```

```
# irrt (maintenance dyalisis) = No
merged_data_all <- merged_data_all %>%
  mutate(irrt = ifelse(irrt == 1 & dialysis == 1, 0, irrt))

# pex
merged_data_all$pex <- ifelse(merged_data_all$pex == "Yes", 1,
                              ifelse(merged_data_all$pex == "No", 0, NA))

# pmx
merged_data_all$pmx <- ifelse(merged_data_all$pmx == "Yes", 1,
                              ifelse(merged_data_all$pmx == "No", 0, NA))

# aids
merged_data_all$aids <- ifelse(merged_data_all$aids == "Yes", 1,
                               ifelse(merged_data_all$aids == "No", 0, NA))

# heart_failure
merged_data_all$heart_failure <- ifelse(merged_data_all$heart_failure == "Yes", 1,
                                        ifelse(merged_data_all$heart_failure == "No",
0, NA))

# resp_failure
merged_data_all$resp_failure <- ifelse(merged_data_all$resp_failure == "Yes", 1,
                                       ifelse(merged_data_all$resp_failure == "No",
0, NA))

# liver_failure
merged_data_all$liver_failure <- ifelse(merged_data_all$liver_failure == "Yes", 1,
                                        ifelse(merged_data_all$liver_failure == "No",
0, NA))

# liver_cirrhosis
merged_data_all$liver_cirrhosis <- ifelse(merged_data_all$liver_cirrhosis == "Yes", 1,
                                          ifelse(merged_data_all$liver_cirrhosis ==
"No", 0, NA))
```

```
# aml
merged_data_all$aml <- ifelse(merged_data_all$aml == "Yes", 1,
                              ifelse(merged_data_all$aml == "No", 0, NA))

# lymphoma
merged_data_all$lymphoma <- ifelse(merged_data_all$lymphoma == "Yes", 1,
                                   ifelse(merged_data_all$lymphoma == "No", 0,
NA))

# aml + lymphoma = hemat_malig
merged_data_all <- merged_data_all %>%
  mutate(
    hemat_malig = ifelse(aml == 0 & lymphoma == 0, 0,　# aml が 0 かつ lymphoma が 0
のとき hemat_は 0
                         ifelse(aml == 1 | lymphoma == 1, 1, NA))　# aml が 1 または
lymphoma が 1 のとき hemat_は 1
  )

# meta
merged_data_all$meta <- ifelse(merged_data_all$meta == "Yes", 1,
                               ifelse(merged_data_all$meta == "No", 0, NA))

# immunosup
merged_data_all$immunosup <- ifelse(merged_data_all$immunosup == "Yes", 1,
                                    ifelse(merged_data_all$immunosup == "No", 0,
NA))

# aids + immunosup = new_immunosup
merged_data_all <- merged_data_all %>%
  mutate(
    new_immunosup = ifelse(aids == 0 & immunosup == 0, 0,　# aids が 0 かつ
immunosup が 0 のとき new_は 0
                           ifelse(aids == 1 | immunosup == 1, 1, NA)))　# aids が 1 ま
たは immunosup が 1 のとき new_は 1
```

```
# dialysis
merged_data_all$dialysis <- ifelse(merged_data_all$dialysis == "Yes", 1,
                                   ifelse(merged_data_all$dialysis == "No", 0, NA))

# tracheostomized
merged_data_all$tracheostomized <- ifelse(merged_data_all$tracheostomized == "Yes", 1,
                                          ifelse(merged_data_all$tracheostomized ==
"No", 0, NA))

# hfnc
merged_data_all$hfnc <- ifelse(merged_data_all$hfnc == "Yes", 1,
                               ifelse(merged_data_all$hfnc == "No", 0, NA))


# NPPV ,HFNC
merged_data_all <- merged_data_all %>%
  mutate(mec_vent = ifelse(is.na(mec_vent_start_day), 0, 1))


# dob
merged_data_all$dob <- ifelse(merged_data_all$dob == "使用した" | merged_data_all$dob
== "使用していた" ,  1,
                              ifelse(merged_data_all$dob == "使用していない" |
                                       merged_data_all$dob == "0 = DOB"  |
                                       merged_data_all$dob == "¥n", 0, NA))

# doa
merged_data_all$doa <-  ifelse(merged_data_all$doa == "15microg/kg/min を超える", 1,
                               ifelse(merged_data_all$doa == "5microg/kg/min を超え
15microg/kg/min 以下", 1,
                                      ifelse(merged_data_all$doa ==
"5microg/kg/min 以下" | merged_data_all$doa == "5γ 以下" | merged_data_all$doa == "0
＜ DOA ≦ 5"  |  merged_data_all$doa  == "0mirog/kg/min を超え 5microg/kg/min 以
下",  1,
                                             ifelse(merged_data_all$doa == "No" |
merged_data_all$doa == "使用していない", 0, NA ))))
```

```
# nad
merged_data_all$nad <- ifelse(merged_data_all$nad == "0.15γ を超える" |
                              merged_data_all$nad == "0.2microg/kg/min を超える"
|
                              merged_data_all$nad == "0.1microg/kg/min を超える
", 1,
                            ifelse(merged_data_all$nad == "0.1microg/kg/min 以下"
|
                                   merged_data_all$nad == "0.1γ 以下", 1,
                                 ifelse(merged_data_all$nad == "0 = NOR" |
                                        merged_data_all$nad == "使用してい
ない" |
                                        merged_data_all$nad == "使用してい
な¥n い", 0, NA)))

# ad
merged_data_all$ad <- ifelse(merged_data_all$ad == "0.16γ を超える" |
                             merged_data_all$ad == "0.1microg/kg/min を超える" ,
1,
                           ifelse(merged_data_all$ad == "0.1microg/kg/min 以下" |
                                  merged_data_all$ad == "使用した", 1,
                                ifelse(merged_data_all$ad == "0 = ADR" |
                                       merged_data_all$ad == "使用していな
い" |
                                       merged_data_all$ad == "使用していな
い." |
                                       merged_data_all$ad == "使用していな
い¥n0.1microg/kg/min 以下", 0, NA)))


# aki
merged_data_all$aki <- ifelse(merged_data_all$aki == "Yes", 1,
                            ifelse(merged_data_all$aki == "No", 0, NA))
```

```
# re_cabg
merged_data_all$re_cabg <- ifelse(merged_data_all$re_cabg == "Yes", 1,
                                   ifelse(merged_data_all$re_cabg == "No", 0, NA))

# mec_vent_24h
merged_data_all$mec_vent_24h <- ifelse(merged_data_all$mec_vent_24h == "Yes", 1,
                                        ifelse(merged_data_all$mec_vent_24h ==
"No", 0, NA))


# hosp_type
merged_data_all$hosp_type <-  ifelse(merged_data_all$hosp_type == "私立大学", 6,
                                     ifelse(merged_data_all$hosp_type == "公立大学",
5,
                                            ifelse(merged_data_all$hosp_type == "私
立病院", 4,
                                                   ifelse(merged_data_all$hosp_type
== "国立病院", 3,

ifelse(merged_data_all$hosp_type == "公的病院", 2,

ifelse(merged_data_all$hosp_type == "国立大学", 1,

ifelse(merged_data_all$hosp_type == "公立病院",0, NA)))))))

# hospital type
merged_data_all <- merged_data_all %>%
  mutate(academic = ifelse(hosp_type %in% c(1, 5, 6), 0, 1))

# hosp_type
unique(merged_data_all$hosp_type)

# infection
merged_data_all <- merged_data_all  %>%
  mutate(infection = ifelse(disease_code %in% c(201, 210, 212, 213, 308, 313,
                                                404, 501, 502, 503, 504, 1102,
```

1301, 1401, 1406, 1409, 1412, 1904),
1, 0))

main code
#101- 循環器
#201- 呼吸器
#301- 消化器
#401- 神経
#501- 敗血症
#601- 外傷
#701- 代謝
#801- 血液
#901- 生殖泌尿器
#1002 その他の内科
#1101-膠原病・皮膚軟部
#1202-循環器手術
#1301- 呼吸器手術
#1401- 消化器手術
#1501- 神経手術
#1601- 外傷手術
#1701- 泌尿器手術
#1801- 婦人科手術
#1902 整形
#1903 形成・乳腺手術
#1904 軟部組織
#2101- 血液疾患手術
#2201- 代謝手術

diagnosis_code
cardiovascular 1 (100 1200)
gastrointestinal 2 (300 1400)
musculoskeletal 3 (1902)
metabolic_endocrine 4 (700 2200)
neurological 5 (400 1500)
respiratory 6 (200 1300)
trauma 7 (600 1600)

```
# urinary_gynecological 8 (900 1700 1800)
# others 9 (血液や軟部組織感染、分類不能の敗血症、形成、乳腺など)
merged_data_all <- merged_data_all %>%
  mutate(diagnosis_code = case_when(
    (disease_code >= 100 & disease_code < 200) | (disease_code >= 1200 & disease_code
< 1300) ~ "1",
    (disease_code >= 300 & disease_code < 400) | (disease_code >= 1400 & disease_code
< 1500) ~ "2",
    disease_code == 1902 ~ "3",
    (disease_code >= 700 & disease_code < 800) | (disease_code >= 2200 & disease_code
< 2300) ~ "4",
    (disease_code >= 400 & disease_code < 500) | (disease_code >= 1500 & disease_code
< 1600) ~ "5",
    (disease_code >= 200 & disease_code < 300) | (disease_code >= 1300 & disease_code
< 1400) ~ "6",
    (disease_code >= 600 & disease_code < 700) | (disease_code >= 1600 & disease_code
< 1700) ~ "7",
    (disease_code >= 900 & disease_code < 1000) | (disease_code >= 1700 &
disease_code < 1900) ~ "8",
    TRUE ~ "9"
  ))


# num_me_24h
merged_data_all$num_me_24h <- ifelse(merged_data_all$num_me_24h == "有" |
                                     merged_data_all$num_me_24h == "1" |
                                     merged_data_all$num_me_24h == "2" |
                                     merged_data_all$num_me_24h == "12" |
                                     merged_data_all$num_me_24h == "16" |
                                     merged_data_all$num_me_24h == "14" |
                                     merged_data_all$num_me_24h == "1-2" |
                                     merged_data_all$num_me_24h == "3" |
                                     merged_data_all$num_me_24h == " 1 " |
                                     merged_data_all$num_me_24h == "1~2" |
                                     merged_data_all$num_me_24h == "#N/A" |
```

```
                                                        merged_data_all$num_me_24h == "4" |
                                                        merged_data_all$num_me_24h == "11" |
                                                        merged_data_all$num_me_24h == "9" |
                                                        merged_data_all$num_me_24h == "5" , 1,
                                                      ifelse(merged_data_all$num_me_24h == "無" |
                                                               merged_data_all$num_me_24h == "0",
0, NA))


## LOS ICU
# icu admission time
merged_data_all$icu_adm_total_hours <- merged_data_all$icu_adm_day * 24 +
  as.numeric(substr(merged_data_all$icu_adm_time, 1, 2)) +        # 時を加算
  as.numeric(substr(merged_data_all$icu_adm_time, 4, 5)) / 60 +   # 分を時間に換算して
加算
  as.numeric(substr(merged_data_all$icu_adm_time, 7, 8)) / 3600 # 秒を時間に換算して
加算

# icu discharge time
merged_data_all$icu_disc_total_hours <- merged_data_all$icu_disc_day * 24 +
  as.numeric(substr(merged_data_all$icu_disc_time, 1, 2)) +         # 時を加算
  as.numeric(substr(merged_data_all$icu_disc_time, 4, 5)) / 60 +     # 分を時間に換算し
て加算
  as.numeric(substr(merged_data_all$icu_disc_time, 7, 8)) / 3600   # 秒を時間に換算して
加算

# LOS ICU
merged_data_all$icu_duration <- merged_data_all$icu_disc_total_hours -
merged_data_all$icu_adm_total_hours



# admission - ICU
#
class(merged_data_all$adm_day)
class(merged_data_all$icu_adm_day)
# days_between_hosp_icu
```

```
merged_data_all   <- merged_data_all %>%
  mutate(days_between_hosp_icu = icu_adm_day - adm_day)

# GCS
merged_data_all <- merged_data_all %>%
  mutate(gcs = gcs_e + gcs_v + gcs_m)


## intensivist
#
table(merged_data_all$num_fulltime_specialist)
table(merged_data_all$num_fulltime_unspecialist)

# num_full_specialist
merged_data_all <- merged_data_all %>%
  mutate(num_fulltime_specialist = str_replace_all(num_fulltime_specialist, " 7 ", "7"))
#
table(merged_data_all$num_fulltime_specialist)

# factor→character→numbermerged_data_all <- merged_data_all %>%
  mutate(
    num_fulltime_specialist = as.numeric(as.character(num_fulltime_specialist)),
    num_fulltime_unspecialist = as.numeric(as.character(num_fulltime_unspecialist))
  )


# add
merged_data_all <- merged_data_all %>%
  mutate(num_fulltime_intensivist = num_fulltime_specialist + num_fulltime_unspecialist)

#
table(merged_data_all$num_fulltime_intensivist)

# BMI
merged_data_all <- merged_data_all %>%
  mutate(
```

```
    bmi_raw = weight / ((height / 100) * (height / 100)),
    bmi = round(bmi_raw, 1)
  )
```

```
## ABGs
# どうすれば適切に回数がわかるか探る
# ph_1 の欠損を確認 46040 もある
table(is.na(merged_data_all$ph_1))

# ph_1,pao2_1,paco2_1 のすべてが NA である行数 =45006
sum(is.na(merged_data_all$ph_1) & is.na(merged_data_all$pao2_1) &
is.na(merged_data_all$paco2_1))

# ph_1,pao2_1,paco2_1 のすべてが NA であるが、ph_2 以降のいずれか NA ではない行数
=5486
sum(
  is.na(merged_data_all$ph_1) & is.na(merged_data_all$pao2_1) &
is.na(merged_data_all$paco2_1) &
    (!is.na(merged_data_all$ph_2) | !is.na(merged_data_all$pao2_2)
| !is.na(merged_data_all$paco2_2) |
       !is.na(merged_data_all$ph_3) | !is.na(merged_data_all$pao2_3)
| !is.na(merged_data_all$paco2_3) |
       !is.na(merged_data_all$ph_4) | !is.na(merged_data_all$pao2_4)
| !is.na(merged_data_all$paco2_4) |
       !is.na(merged_data_all$ph_5) | !is.na(merged_data_all$pao2_5)
| !is.na(merged_data_all$paco2_5) |
       !is.na(merged_data_all$ph_6) | !is.na(merged_data_all$pao2_6)
| !is.na(merged_data_all$paco2_6)))

# 確かめる
inconsistent_rows <- merged_data_all %>%
  filter(
    is.na(ph_1) & is.na(pao2_1) & is.na(paco2_1) &
```

```
        (!is.na(ph_2) | !is.na(pao2_2) | !is.na(paco2_2) |
           !is.na(ph_3) | !is.na(pao2_3) | !is.na(paco2_3) |
           !is.na(ph_4) | !is.na(pao2_4) | !is.na(paco2_4) |
           !is.na(ph_5) | !is.na(pao2_5) | !is.na(paco2_5) |
           !is.na(ph_6) | !is.na(pao2_6) | !is.na(paco2_6))
  )

print(nrow(inconsistent_rows))   # 5486 例ある
view(inconsistent_rows)

# 0 は NA にする  across を使って一気に
merged_data_all <- merged_data_all %>%
  mutate(across(c(ph_1:ph_6, pao2_1:pao2_6, paco2_1:paco2_6, fio2_1:fio2_6),
                ~ ifelse(. == 0, NA, .)))

# ph_1,pao2_1,paco2_1 のすべてが NA であるが、ph_2 以降のいずれか NA ではない行数
もう一度 ＝3
sum(
  is.na(merged_data_all$ph_1) & is.na(merged_data_all$pao2_1) &
is.na(merged_data_all$paco2_1) &
    (!is.na(merged_data_all$ph_2) | !is.na(merged_data_all$pao2_2)
| !is.na(merged_data_all$paco2_2) |
       !is.na(merged_data_all$ph_3) | !is.na(merged_data_all$pao2_3)
| !is.na(merged_data_all$paco2_3) |
       !is.na(merged_data_all$ph_4) | !is.na(merged_data_all$pao2_4)
| !is.na(merged_data_all$paco2_4) |
       !is.na(merged_data_all$ph_5) | !is.na(merged_data_all$pao2_5)
| !is.na(merged_data_all$paco2_5) |
       !is.na(merged_data_all$ph_6) | !is.na(merged_data_all$pao2_6)
| !is.na(merged_data_all$paco2_6)))

#  確かめる
inconsistent_rows <- merged_data_all %>%
  filter(
    is.na(ph_1) & is.na(pao2_1) & is.na(paco2_1) &
      (!is.na(ph_2) | !is.na(pao2_2) | !is.na(paco2_2) |
```

```
            !is.na(ph_3) | !is.na(pao2_3) | !is.na(paco2_3) |
            !is.na(ph_4) | !is.na(pao2_4) | !is.na(paco2_4) |
            !is.na(ph_5) | !is.na(pao2_5) | !is.na(paco2_5) |
            !is.na(ph_6) | !is.na(pao2_6) | !is.na(paco2_6))
  )

print(nrow(inconsistent_rows))  # 3 例ある
view(inconsistent_rows)  # 、FiO2_1 だけ入ってるのが 3 例. 3 例は測定したことにする



# num_abg
merged_data_all <- merged_data_all %>%
  mutate(
    num_abg = case_when(
      rowSums(!is.na(select(., ph_6, pao2_6, paco2_6, fio2_6))) > 0 ~ 6,
      rowSums(!is.na(select(., ph_5, pao2_5, paco2_5, fio2_5))) > 0 ~ 5,
      rowSums(!is.na(select(., ph_4, pao2_4, paco2_4, fio2_4))) > 0 ~ 4,
      rowSums(!is.na(select(., ph_3, pao2_3, paco2_3, fio2_3))) > 0 ~ 3,
      rowSums(!is.na(select(., ph_2, pao2_2, paco2_2, fio2_2))) > 0 ~ 2,
      rowSums(!is.na(select(., ph_1, pao2_1, paco2_1, fio2_1))) > 0 ~ 1,
      TRUE ~ 0))  # すべてが NA の場合



# delete
# lack of nurse information
merged_data_all  <- merged_data_all %>%
  mutate(num_fulltime_nurse = as.numeric(as.character(num_fulltime_nurse)))

merged_data_all %>%
  filter(is.na(num_fulltime_nurse)) %>%
  count(id)

# lac of doctor information
merged_data_all %>%
```

```
  filter(is.na(num_fulltime_intensivist)) %>%
  count(id)

# id
excluded_ids <- c(140, 203, 249, 263, 316, 319, 129, 363)

merged_data_all %>%
  filter(id %in% excluded_ids) %>%
  count(id)


# delete 8 institution 324396→310424 (-13972)
merged_data_all <- merged_data_all %>%
  filter(!(id %in% c(140, 203, 249, 263, 316, 319, 129, 363)))
324396-310424

# delete children hospital
# children hospital
df_pediatric_ids <- merged_data_all %>%
  group_by(id) %>%
  summarise(
    total = n(),
    under15 = sum(age < 15, na.rm = TRUE)
  ) %>%
  mutate(under15_rate = under15 / total) %>%
  filter(under15_rate > 0.5)

# delete     310424→303093 (-7331)
merged_data_all <- merged_data_all %>%
  filter(!(id %in% c(127, 301, 312, 313, 320)))
310424-303093


# number of institutions
length(unique(df_patient$"施設固有 ID"))
length(unique(merged_data_all$id))
```

```
## only after JIPAD 3.0

# plt_min, lac, hfnc, doa, dob, nad, ad 全て NA の症例を除外(いずれかがあれば 3.0 だと言える) 303093→248540 (-54553)
merged_data_all <- merged_data_all %>%
  filter(!(is.na(plt_min) & is.na(lac) & is.na(hfnc) &
             is.na(doa) & is.na(dob) & is.na(nad) & is.na(ad)))

303093-248540

#
length(unique(merged_data_all$id))




## inclusion
# age 248540-240078 (-8462)
merged_data_all <- merged_data_all[merged_data_all$age >= 16, ]
248540-240078

# length of ICU 240078-134141 -(105937)
merged_data_all <- merged_data_all [merged_data_all$icu_duration >= 24, ]
240078-134141

# technical 134141 - 134047 = 94(手技で入って 1 日以上いた人がいるってことだけどフローでは<24h での除外とする)
merged_data_all <- merged_data_all %>%
  filter(type_adm != 2)
134141 - 134047

#sum -106031
240078 - 134047
```

```
# A-line 134047 - 126120(-7927)
merged_data_all <- merged_data_all [merged_data_all$a_line >= 1, ]
134047 - 126120

# re-admission 126120 - 118420(-7700)
merged_data_all <- merged_data_all %>%  filter(re_adm == 0)
126120 - 118420




# df re-cleaning
#delete
#
colnames(merged_data_all)

#
columns_to_remove_2 <- c(
  "pr_max",    "pr_min",    "sbp_max",   "sbp_min",
  "mbp_max",   "mbp_min",   "dbp_max",   "dbp_min",
  "bt_max",    "bt_min",    "rr_max",    "rr_min",
  "ht_max",    "ht_min",    "wbc_max",   "wbc_min",
  "cre_min",   "bun_max",   "urin_vol", "na_max",
  "na_min",    "k_max",     "k_min",     "alb_max",
  "alb_min",   "bil_max",   "bs_max",    "bs_min",
  "adm_hash", "patient_hash"
)


#delete
merged_data_all <- merged_data_all[, -which(names(merged_data_all) %in%
columns_to_remove_2)]
```

```
###EDA###----

#
glimpse(merged_data_all)


# sex, hosp_outcome, new_type_adm, hemat_malig, new_immunosup, mec_vent, academic,
# infection, diagnosis_code, ecmo, re_adm, cv_line, nppv, iabp, va_ecmo, vv_ecmo,
# irrt, crrt, pex, pmx, ohter_purification, heart_failure, resp_failure,
# liver_failure, liver_cirrhosis, meta, dialysis,
# mec_vent_24h, hfnc, em_call を因子型にする
merged_data_all$sex <- factor(merged_data_all$sex)
merged_data_all$hosp_outcome <- factor(merged_data_all$hosp_outcome)
merged_data_all$new_type_adm <- factor(merged_data_all$new_type_adm)
merged_data_all$hemat_malig <- factor(merged_data_all$hemat_malig)
merged_data_all$new_immunosup <- factor(merged_data_all$new_immunosup)
merged_data_all$mec_vent <- factor(merged_data_all$mec_vent)
merged_data_all$academic <- factor(merged_data_all$academic)
merged_data_all$infection <- factor(merged_data_all$infection)
merged_data_all$diagnosis_code <- factor(merged_data_all$diagnosis_code)
merged_data_all$ecmo <- factor(merged_data_all$ecmo)
merged_data_all$re_adm <- factor(merged_data_all$re_adm)
merged_data_all$cv_line <- factor(merged_data_all$cv_line)
merged_data_all$nppv <- factor(merged_data_all$nppv)
merged_data_all$iabp <- factor(merged_data_all$iabp)
merged_data_all$va_ecmo <- factor(merged_data_all$va_ecmo)
merged_data_all$vv_ecmo <- factor(merged_data_all$vv_ecmo)
merged_data_all$irrt <- factor(merged_data_all$irrt)
merged_data_all$crrt <- factor(merged_data_all$crrt)
merged_data_all$pex <- factor(merged_data_all$pex)
merged_data_all$pmx <- factor(merged_data_all$pmx)
merged_data_all$other_purification <- factor(merged_data_all$other_purification)
merged_data_all$heart_failure <- factor(merged_data_all$heart_failure)
merged_data_all$resp_failure <- factor(merged_data_all$resp_failure)
merged_data_all$liver_failure <- factor(merged_data_all$liver_failure)
merged_data_all$liver_cirrhosis <- factor(merged_data_all$liver_cirrhosis)
```

```
merged_data_all$meta <- factor(merged_data_all$meta)
merged_data_all$dialysis <- factor(merged_data_all$dialysis)
merged_data_all$mec_vent_24h <- factor(merged_data_all$mec_vent_24h)
merged_data_all$hfnc <- factor(merged_data_all$hfnc)
merged_data_all$id <- factor(merged_data_all$id)
merged_data_all$year <- factor(merged_data_all$year)
merged_data_all$em_call <- factor(merged_data_all$em_call)

#
glimpse(merged_data_all)


# factor → character → numeric
merged_data_all <- merged_data_all %>%
  mutate(
    num_ecmo = as.numeric(as.character(num_ecmo)),
    num_crrt = as.numeric(as.character(num_crrt)),
    num_bed_icu = as.numeric(as.character(num_bed_icu)),
    area_bed = as.numeric(as.character(area_bed)),
    area_open = as.numeric(as.character(area_open)),
    area_closed = as.numeric(as.character(area_closed)),
    num_fulltime_specialist = as.numeric(as.character(num_fulltime_specialist)),
    num_fulltime_unspecialist = as.numeric(as.character(num_fulltime_unspecialist)),
    num_fulltime_over5year = as.numeric(as.character(num_fulltime_over5year)),
    num_specialist = as.numeric(as.character(num_specialist)),
    num_rotater = as.numeric(as.character(num_rotater)),
    num_resident = as.numeric(as.character(num_resident)),
    num_fulltime_nurse = as.numeric(as.character(num_fulltime_nurse)),
    num_nurse_certified_cc = as.numeric(as.character(num_nurse_certified_cc)),
    num_nurse_certified_em = as.numeric(as.character(num_nurse_certified_em)),
    num_nurse_specialized = as.numeric(as.character(num_nurse_specialized)),
    num_me_hosp = as.numeric(as.character(num_me_hosp)),
    num_me_24h = as.numeric(as.character(num_me_24h)),
    num_fulltime_me = as.numeric(as.character(num_fulltime_me)),
    time_me = as.numeric(as.character(time_me)),
    num_fulltime_pharm = as.numeric(as.character(num_fulltime_pharm)),
```

```
    time_pharm = as.numeric(as.character(time_pharm)),
    num_fulltime_intensivist = as.numeric(as.character(num_fulltime_intensivist)),
    num_bed_hosp = as.numeric(as.character(num_bed_hosp)),
    icu_adm_total_hours = as.numeric(as.character(icu_adm_total_hours)),
    icu_disc_total_hours = as.numeric(as.character(icu_disc_total_hours)),
    icu_duration = as.numeric(as.character(icu_duration)),
    days_between_hosp_icu = as.numeric(as.character(days_between_hosp_icu)),
    gcs = as.numeric(as.character(gcs))
  )

#
glimpse(merged_data_all)


# summary
summary(merged_data_all)



#
library(Hmisc)

describe(merged_data_all[, c("height")])


# weight の min 0 は確実にはずれ値、max 217 は身長と照らし合わせたり分布を診たりすると意外と有り得そう
options(max.print = 10000)　# 1 万行まで表示可能に
table(merged_data_all$weight) #table で確認

merged_data_all <- merged_data_all %>%
  mutate(weight = ifelse(weight %in% c(0, 1), NA, weight)) #  はずれ値を NA に（0kg、1kg)

#177cm/10kg と 173cm/10kg と 159cm/15kg と 158cm/18kg ははずれ値
merged_data_all[rownames(merged_data_all) %in% c("62148", "98229", "98572",
```

```
"58151"), "weight"] <- NA



#height の min 0 も確実にはずれ値
merged_data_all <- merged_data_all %>%
  mutate(height = ifelse(height %in% c(0, 1), NA, height))  #はずれ値を NA に（0cm)
#<100cm は多くは年齢、体重と照らし合わせても 100 の位を忘れたものと思われる
# 71cm/8kg がいるのでまずは≦70 を修正
merged_data_all <- merged_data_all %>%
  mutate(height = ifelse(height <= 70, height + 100, height))
#90cm/33kg は NA に
merged_data_all[rownames(merged_data_all) %in% c("3969"), "height"] <- NA
# 75-85cm を修正
merged_data_all <- merged_data_all %>%
  mutate(height = ifelse(height >= 75 & height <= 85, height + 100, height))
#94cm/48kg,94cm/28kg,96cm/42kg,97cm/50kg,97cm/59kg は NA に
merged_data_all[rownames(merged_data_all) %in% c("177393", "254736", "277381",
"58480", "58746", ""), "height"] <- NA
#98-100cm は明らかにおかしいので NA に。2 人 18 歳と 19 歳はあり得るので残す
merged_data_all <- merged_data_all %>%
  mutate(
    height = ifelse(height >= 98 & height <= 100 & age >= 20, NA, height),
    weight = ifelse(height >= 98 & height <= 100 & age >= 20, NA, weight))
#109cm 74kg は NA に
merged_data_all[rownames(merged_data_all) %in% c("292669"), "height"] <- NA

# apache2 = 0
# APACHE II スコアが 0 の行を抽出→他の項目を見る限り妥当
apache_0_rows <- merged_data_all %>%
  filter(apache2 == 0)

print(apache_0_rows)

# 血小板 0 は血液疾患か
# `plt_min` が 0 の行の `disease_code` を抽出
```

```
disease_codes <- merged_data_all %>%
  filter(plt_min == 0) %>%
  select(disease_code)

print(disease_codes)

# GCS2 = NA
merged_data_all <- merged_data_all %>%
  mutate(gcs = ifelse(gcs %in% c(2), NA, gcs))




# lack
colSums(is.na(merged_data_all))  # 各列ごとの欠損値の数を表示

na_rows <- merged_data_all %>%
  filter(is.na(hosp_outcome)) # それを抽出


## 大事な列で NA を含む行を削除 118420→118419
# 退院時転帰 hosp_outcome
merged_data_all <- merged_data_all %>%
  filter(!is.na(hosp_outcome))

# 2018 年度に 2 人いて、1 人は 10 歳で除外済、もう一人が欠損しているようだ
df_patient[is.na(df_patient$退院時転帰), ]


# jrod_mortality → %
merged_data_all <- merged_data_all %>%
  mutate(jrod_mortality_100 = jrod_mortality * 100)


# BMI の 0 は NA に  <7.5 も NA に
filtered_data <- merged_data_all %>%
  filter(bmi <= 10) %>%  # bmi が 10 以下の行を抽出
```

```
  select(weight, height, bmi, age)   # weight と height の列のみを選択

merged_data_all <- merged_data_all %>%
  mutate(bmi = ifelse(bmi <7.5, NA, bmi))



## ABGs cleaning   ph, pf, paco2
# ph_1 ~ ph_6
ph_cols <- grep("^ph_[1-6]$", names(merged_data_all), value = TRUE)

# paco2_1 ~ paco2_6
paco2_cols <- grep("^paco2_[1-6]$", names(merged_data_all), value = TRUE)

# pao2_1 ~ pao2_6
pao2_cols <- grep("^pao2_[1-6]$", names(merged_data_all), value = TRUE)

# fio2_1 ~ fio2_6
fio2_cols <- grep("^fio2_[1-6]$", names(merged_data_all), value = TRUE)

# ph_low, ph_high
merged_data_all <- merged_data_all %>%
  mutate(
    ph_low = apply(select(., all_of(ph_cols)), 1, function(x) if(all(is.na(x))) NA else min(x,
na.rm = TRUE)),
    ph_high = apply(select(., all_of(ph_cols)), 1, function(x) if(all(is.na(x))) NA else
max(x, na.rm = TRUE))
  )

# pao2_x / fio2_x = pf_x
for (i in 1:6) {
  pao2_name <- paste0("pao2_", i)
  fio2_name <- paste0("fio2_", i)
  pf_name <- paste0("pf_", i)

  if (pao2_name %in% names(merged_data_all) & fio2_name %in%
names(merged_data_all)) {
```

```
    merged_data_all <- merged_data_all %>%
      mutate(!!pf_name := get(pao2_name) / get(fio2_name))
  }
}

# pf_1 ~ pf_6
pf_cols <- grep("^pf_[1-6]$", names(merged_data_all), value = TRUE)

# pf_low, pf_high
merged_data_all <- merged_data_all %>%
  mutate(
    pf_low = apply(select(., all_of(pf_cols)), 1, function(x) if(all(is.na(x))) NA else min(x,
na.rm = TRUE)),
    pf_high = apply(select(., all_of(pf_cols)), 1, function(x) if(all(is.na(x))) NA else max(x,
na.rm = TRUE))
  )

# paco2_low, paco2_high
merged_data_all <- merged_data_all %>%
  mutate(
    paco2_low = apply(select(., all_of(paco2_cols)), 1, function(x) if(all(is.na(x))) NA else
min(x, na.rm = TRUE)),
    paco2_high = apply(select(., all_of(paco2_cols)), 1, function(x) if(all(is.na(x))) NA
else max(x, na.rm = TRUE))
  )

#
df_abg_data <- merged_data_all %>%
  select(ph_1:ph_6,fio2_1:fio2_6, pao2_1:pao2_6, paco2_1:paco2_6, ph_low, ph_high,
pf_low, pf_high, paco2_low, paco2_high)




## categorize
```

```
# ph_low
merged_data_all <- merged_data_all %>%
  mutate(
    ph_low_categ = case_when(
      is.na(ph_low) ~ 0,
      ph_low < 7.15 ~ 1,
      ph_low >= 7.15 & ph_low < 7.25 ~ 2,
      ph_low >= 7.25 & ph_low < 7.35 ~ 3,
      ph_low >= 7.35 & ph_low <= 7.45 ~ 4,
      ph_low > 7.45 ~ 5
    )
  )

# paco2_high
merged_data_all <- merged_data_all %>%
  mutate(
    paco2_high_categ = case_when(
      is.na(paco2_high) ~ 0,
      paco2_high < 35 ~ 1,
      paco2_high >= 35 & paco2_high <= 45 ~ 2,
      paco2_high > 45 & paco2_high <= 55 ~ 3,
      paco2_high > 55 & paco2_high <= 60 ~ 4,
      paco2_high >60 ~ 5
    )
  )



# reference = normal
# ph_low
merged_data_all <- merged_data_all %>%
  mutate(
    ph_low_categ = recode(
      ph_low_categ,
      `0` = 5,
      `4` = 0,
```

```
      `5` = 4
    )
  )


# paco2_high
merged_data_all <- merged_data_all %>%
  mutate(
    paco2_high_categ = recode(
      paco2_high_categ,
      `0` = 5,
      `2` = 0,
      `3` = 2,
      `4` = 3,
      `5` = 4
    )
  )


# pf
merged_data_all <- merged_data_all %>%
  mutate(
    pf_categ = case_when(
      pf_low >= 400 ~ 0,
      pf_low >= 300 ~ 1,
      pf_low >= 200 ~ 2,
      pf_low >= 100 ~ 3,
      pf_low < 100 ~ 4,
      is.na(pf_low) ~ 5
    ))

#
table(merged_data_all$ph_categ)
table(merged_data_all$paco2_categ)
table(merged_data_all$pf_categ)
```

```
# lactate categorize
# summary
summary(merged_data_all$lac)

# look
lac_outcome_df <- merged_data_all %>%
  select(lac, hosp_outcome)

# lac≧30 → mmol/l
merged_data_all <- merged_data_all %>%
  mutate(lac = ifelse(lac>=30, lac/9, lac))

# summary
summary(merged_data_all$lac)

# with my SOFA　0~1, 2~3, 4~5, 6~9, 10~
merged_data_all <- merged_data_all %>%
  mutate(
    lac_categ = case_when(
      lac <2 ~ 0,
      lac <4 ~ 1,
      lac <6 ~ 2,
      lac <10 ~ 3,
      lac >=10 ~4,
      is.na(lac) ~ 5
    ))


# factor
merged_data_all$lac_categ <- as.factor(merged_data_all$lac_categ)
merged_data_all$ph_low_categ <- as.factor(merged_data_all$ph_low_categ)
merged_data_all$pf_categ <- as.factor(merged_data_all$pf_categ)
merged_data_all$paco2_high_categ <- as.factor(merged_data_all$paco2_high_categ)
merged_data_all$lac_categ <- as.factor(merged_data_all$lac_categ)
```

```
## ABGs standardization----

# delete lack

# fill lack
# sex NA = 1
merged_data_all[is.na(merged_data_all$sex), ]

merged_data_all <- merged_data_all %>%
  mutate(sex = as.numeric(as.character(sex)),
         sex = if_else(is.na(sex), 1, sex))

table(merged_data_all$sex)

# cvc NA = 0
merged_data_all[is.na(merged_data_all$cv_line), ]

merged_data_all <- merged_data_all %>%
  mutate(cv_line = as.numeric(as.character(cv_line)),
         cv_line = if_else(is.na(cv_line), 0, cv_line))



# aki NA = 0
merged_data_all <- merged_data_all %>%
  mutate(aki = as.numeric(as.character(aki)),
         aki = if_else(is.na(aki), 0, aki))

#
merged_data_all[is.na(merged_data_all$new_type_adm), ]

merged_data_all["12273", "new_type_adm"] <- 1
merged_data_all["110113", "new_type_adm"] <- 1
```

```
# em_call NA = 1　em_call =　0　(not emergency operation)
merged_data_all[is.na(merged_data_all$em_call), ]
merged_data_all$em_call <- as.numeric(as.character(merged_data_all$em_call))
merged_data_all$em_call[is.na(merged_data_all$em_call)] <- 0
merged_data_all$em_call <- factor(merged_data_all$em_call)


# count
missing_counts <- merged_data_all %>%
  summarise(across(c(age, sex, bmi, cv_line, heart_failure, resp_failure, liver_cirrhosis,
                     meta, new_immunosup, dialysis, apache3, infection, lac_categ, aki,
mec_vent_24h,
                     new_type_adm, days_between_hosp_icu, diagnosis_code, em_call,
                     ph_low_categ, pf_categ, paco2_high_categ,
                     num_bed_hosp, num_bed_icu, academic,
                     num_fulltime_intensivist, num_fulltime_nurse),
                   ~ sum(is.na(.)))) %>%
  t() %>%
  as.data.frame() %>%
  tibble::rownames_to_column(var = "variable")  # ←ここで行名を列に変換


# delete 118419 - 1175446(-873;bmi)
merged_data_all <- merged_data_all %>%
  drop_na(age, sex, bmi, cv_line, heart_failure, resp_failure, liver_cirrhosis,
          meta, new_immunosup, dialysis, apache3, infection, lac_categ, aki,
mec_vent_24h,
          new_type_adm, days_between_hosp_icu, diagnosis_code,
          ph_low_categ, pf_categ, paco2_high_categ,
          num_bed_hosp, num_bed_icu, academic,
          num_fulltime_intensivist, num_fulltime_nurse)

118419 - 117546
```

```
# institution
length(unique(merged_data_all$id))


## dr. ns. standardizition
# n.dr.
merged_data_all <- merged_data_all %>%
  mutate(doctor_icubeds_ratio = num_fulltime_intensivist/num_bed_icu)

# n.ns.
merged_data_all <- merged_data_all %>%
  mutate(nurse_icubeds_ratio = num_fulltime_nurse/num_bed_icu)



# prediction ABGs---
model_linear_pred_num_abg <- glm(
  num_abg ~ age + sex + bmi + cv_line +
    heart_failure + resp_failure + liver_cirrhosis +
    meta + new_immunosup + dialysis + apache3 +
    infection + em_call + lac_categ + aki + mec_vent_24h +
    new_type_adm + days_between_hosp_icu + diagnosis_code +
    ph_low_categ + pf_categ + paco2_high_categ,
  family = gaussian(link = "identity"),
  na.action = na.exclude,
  data = merged_data_all,
)

summary(model_linear_pred_num_abg)

exp(coef(model_linear_pred_num_abg))

#
install.packages("broom")
library(broom)
```

```
#
linear_pred_df <- tidy(model_linear_pred_num_abg, conf.int = TRUE)
write.csv(linear_pred_df, file = "linear_pred_df.csv", row.names = FALSE)
```

```
# ----
# 1. matrix
X <- model.matrix(model_linear_pred_num_abg, data = merged_data_all)

# 2. beta
beta <- coef(model_linear_pred_num_abg)

# 3.
merged_data_all$pred_num_abg <- as.vector(X %*% beta)  # 行列の積を計算

# mean
merged_data_all<- merged_data_all %>%
  group_by(id) %>%
  mutate(pred_num_abg_avg = mean(pred_num_abg, na.rm = TRUE))


# mean
merged_data_all <- merged_data_all %>%
  group_by(id) %>%
  mutate(num_abg_avg = mean(num_abg, na.rm = TRUE))


# plot
ggplot(merged_data_all, aes(x = num_abg_avg, y = pred_num_abg_avg)) +
  geom_point(color = "black") +  # データポイント
  geom_abline(slope = 1, intercept = 0, linetype = "solid", color = "gray") +  # y = x の基
```

```
準線
  labs(title = "",
       x = "Actual number of arterial blood gas analysis",
       y = "Predicted number of arterial blood gas analysis") +
  scale_x_continuous(limits = c(0, 6), breaks = seq(0, 6, by = 1)) +  # X 軸の範囲と 1 刻
みの目盛設定
  scale_y_continuous(limits = c(0, 7), breaks = seq(0, 6, by = 1)) +  # Y 軸の範囲と 1 刻
みの目盛設定
  theme_minimal() +
  theme(panel.grid.minor = element_blank())  # 0.5 刻みの補助線を消す


## sort----

# df cleaning
for_barplot <- merged_data_all %>%
  select(id, num_abg_avg) %>%
  distinct() # 重複した行を削除

##plot
# ggplot2 package
library(ggplot2)

# sort
ggplot(for_barplot, aes(x = reorder(id, num_abg_avg), y = num_abg_avg)) +
  geom_col(fill = "gray", color = "gray", width = 0.3) +  # 幅を 0.8 にして間隔を空ける
  labs(x = "Number of ICUs",
       y = "Average number of ABGs within the first 24 hours (up to 6)",
       title = "") +
  theme_classic() +
  scale_x_discrete(expand = c(0, 0)) +      # X 軸の間隔を統一
  theme(axis.text.x = element_text(size = 10),  # X 軸ラベルのサイズを調整
        axis.ticks.x = element_blank()) +  # X 軸の目盛りを消す
  scale_x_discrete(labels = seq(20, 100, by = 20),
                   breaks = levels(reorder(for_barplot$id,
for_barplot$num_abg_avg))[seq(20, 100, by = 20)]) +  # `+` に修正
```

```
  scale_y_continuous(expand = c(0, 0))  # Y 軸の 0 ラインを X 軸に密着


# standardization  num_abg_avg_ratio----
merged_data_all <- merged_data_all %>%
  mutate(num_abg_avg_ratio = num_abg_avg/pred_num_abg_avg)

# 1. num_abg_avg_ratio sort
sorted_merged_data_all <- merged_data_all %>%
  select(id, num_abg_avg_ratio) %>%
  distinct() %>%  #  重複を除く
  arrange(num_abg_avg_ratio)

## categorize

# num_abg_avg_ratio tertile
quantiles <- quantile(sorted_merged_data_all$num_abg_avg_ratio, probs = c(1/3, 2/3),
na.rm = TRUE)

sorted_merged_data_all$tertile <- cut(sorted_merged_data_all$num_abg_avg_ratio,
                                      breaks = c(-Inf, quantiles[1], quantiles[2], Inf),
                                      labels = c("Low", "Medium", "High"),
                                      include.lowest = TRUE)

#
table(sorted_merged_data_all$tertile)

# low = 1, Medium = 2, High = 3
sorted_merged_data_all$tertile <- ifelse(sorted_merged_data_all$tertile == "High", 3,
                                         ifelse(sorted_merged_data_all$tertile ==
"Medium", 2,
                                                ifelse(sorted_merged_data_all$tertile
== "Low", 1, NA)))


table(sorted_merged_data_all$tertile)
```

```
# merge with merged_data_all
merged_data_all <- merge(merged_data_all, sorted_merged_data_all, by= c("id"))

# tertle→ factor
merged_data_all$tertile <- as.factor(merged_data_all$tertile)

# plot
library(ggplot2)

# shapes colors
shapes <- c("1" = 16, "2" = 17, "3" = 15)   # 1 → ●(16), 2 → ▲(17), 3 → ■(15)
colors <- c("1" = "black", "2" = "blue", "3" = "red")   # 1 → 黒, 2 → 青, 3 → 赤

# levels
merged_data_all$tertile_label <- factor(
  merged_data_all$tertile,
  levels = c(1, 2, 3),
  labels = c("tertile1", "tertile2", "tertile3")
)

# plot
ggplot(merged_data_all, aes(x = num_abg_avg, y = pred_num_abg_avg, shape =
as.factor(tertile), color = as.factor(tertile))) +
  geom_point(size = 2) +
  geom_abline(slope = 1, intercept = 0, linetype = "solid", color = "gray") +
  scale_shape_manual(
    name = "Tertile",
    values = shapes,
    labels = c("Low SNABGs", "Medium SNABGs", "High SNABGs")
  ) +
  scale_color_manual(
    name = "Tertile",
    values = colors,
    labels = c("Low SNABGs", "Medium SNABGs", "High SNABGs")
  ) +
```

```
  labs(
    title = "",
    x = "Actual number of ABGs within the first 24 hours (up to 6)",
    y = "Predicted number of ABGs within the first 24 hours"
  ) +
  scale_x_continuous(limits = c(0, 6), breaks = seq(0, 6, by = 1)) +
  scale_y_continuous(limits = c(0, 6.2), breaks = seq(0, 6, by = 1)) +
  theme_minimal() +
  theme(panel.grid.minor = element_blank())



## 予後情報追加----

# 施設ごとの ICU 死亡率 icu_mortality_facility 列を作る
library(dplyr)

icu_mortality_facility <- merged_data_all %>%
  group_by(id) %>%
  summarise(icu_mortality_facility = mean(icu_outcome == 0, na.rm = TRUE) * 100)

merged_data_all <- merged_data_all %>%
  left_join(icu_mortality_facility, by = "id")



#  hosp_mortality_facility
hosp_mortality_facility <- merged_data_all %>%
  group_by(id) %>%
  summarise(hosp_mortality_facility = mean(hosp_outcome == 1, na.rm = TRUE) * 100)

merged_data_all <- merged_data_all %>%
  left_join(hosp_mortality_facility, by = "id")


#  icu_mortality_tertile
```

```
tertile_icu_mortality <- merged_data_all %>%
  group_by(tertile) %>%
  summarise(icu_mortality_tertile = mean(icu_outcome == 0, na.rm = TRUE) * 100)

merged_data_all <- merged_data_all %>%
  left_join(tertile_icu_mortality, by = "tertile")


# hosp_mortality_tertile
tertile_hosp_mortality <- merged_data_all %>%
  group_by(tertile) %>%
  summarise(hosp_mortality_tertile = mean(hosp_outcome == 1, na.rm = TRUE) * 100)

merged_data_all <- merged_data_all %>%
  left_join(tertile_hosp_mortality, by = "tertile")


# icu_days_facility(mean)
icu_mean_by_facility <- merged_data_all %>%
  group_by(id) %>%
  summarise(icu_days_facility = mean(icu_adm_day, na.rm = TRUE))

merged_data_all <- merged_data_all %>%
  left_join(icu_mean_by_facility, by = "id")



# hosp_days_facility (mean)
hosp_mean_by_facility <- merged_data_all %>%
  group_by(id) %>%
  summarise(hosp_days_facility = mean(disc_day, na.rm = TRUE))

merged_data_all <- merged_data_all %>%
  left_join(hosp_mean_by_facility, by = "id")
```

```
### figure table###----

## table1
install.packages("tableone")
library(tableone)

#
colnames(merged_data_all)

# N
merged_data_all <- merged_data_all %>%
   group_by(id) %>%
   mutate(num_patient = n()) %>%
   ungroup()



# patient data
tbl_1_patient <- CreateTableOne(
   vars = c("age", "sex", "bmi", "cv_line",
                "heart_failure", "resp_failure", "liver_cirrhosis",
                "meta", "new_immunosup", "dialysis", "sofa", "apache3",
                "infection", "lac_categ", "aki", "mec_vent_24h",
                "new_type_adm", "em_call", "days_between_hosp_icu", "diagnosis_code",
                "ph_high_categ", "ph_low_categ", "pf_categ", "paco2_high_categ" ,
"paco2_low_categ",
                "num_bed_hosp", "num_bed_icu", "academic",
                "num_fulltime_intensivist" , "doctor_icubeds_ratio",
                "num_fulltime_nurse", "nurse_icubeds_ratio", "tertile",
                "icu_adm_day", "disc_day", "icu_outcome", "hosp_outcome"),

   strata = "tertile",
   factorVars = c("sex", "cv_line",
                        "heart_failure", "resp_failure", "liver_cirrhosis",
                        "meta", "new_immunosup", "dialysis",
                        "infection", "lac_categ", "aki", "mec_vent_24h",
```

```
                    "ph_high_categ" , "ph_low_categ", "pf_categ", "paco2_high_categ" ,
"paco2_low_categ",
                    "new_type_adm", "em_call", "diagnosis_code", "academic",
                    "icu_outcome", "hosp_outcome"),

  data = merged_data_all)

print(tbl_1_patient, nonnormal = c("age", "bmi", "sofa", "apache3",
                                   "num_bed_hosp", "num_bed_icu",
                                   "num_fulltime_intensivist", "doctor_icubeds_ratio",
                                   "num_fulltime_nurse", "nurse_icubeds_ratio,",
                                   "days_between_hosp_icu",
                                   "icu_adm_day", "disc_day"
), missing = TRUE, digits = 2)  # 小数点以下 2 桁表示



# SNABG setting
merged_data_all %>%
  group_by(tertile) %>%
  summarise(
    summary_str = paste0(
      round(median(num_abg_avg_ratio.x, na.rm = TRUE), 2),
      " [",
      round(quantile(num_abg_avg_ratio.x, 0.25, na.rm = TRUE), 2),
      "–",
      round(quantile(num_abg_avg_ratio.x, 0.75, na.rm = TRUE), 2),
      "]"
    )
  )



# institution data
# delete
merged_data_all_institution <- merged_data_all %>%
```

```
   group_by(id) %>%
   summarise(across(everything(), first))

# check
table(merged_data_all$num_bed_hosp)
table(merged_data_all$num_bed_icu)
table(merged_data_all$num_fulltime_intensivist)
table(merged_data_all$num_fulltime_nurse)

# institution tbl1
tbl_1_institution <- CreateTableOne(
   vars = c("num_bed_hosp", "num_bed_icu", "academic",
                "num_fulltime_intensivist", "num_fulltime_nurse",
                "doctor_icubeds_ratio", "nurse_icubeds_ratio", "num_patient",
                "icu_mortality_facility", "hosp_mortality_facility", "icu_adm_day", "disc_day"
   ),
   strata = "tertile",
   factorVars = "academic",
   data = merged_data_all_institution
)

print(tbl_1_institution, nonnormal = c("num_bed_hosp", "num_bed_icu",
                                                       "num_fulltime_intensivist",
"num_fulltime_nurse",
                                                       "doctor_icubeds_ratio", "nurse_icubeds_ratio",
"num_patient",
                                                       "icu_mortality_facility",
"hosp_mortality_facility", "icu_adm_day", "disc_day"),
         missing = TRUE)



## table1 before standardization
# patient
tbl_1_patient_pre <- CreateTableOne(
   vars = c("age", "sex", "bmi", "cv_line",
```

```
                    "heart_failure", "resp_failure", "liver_cirrhosis",
                    "meta", "new_immunosup", "dialysis", "sofa", "apache3",
                    "infection", "lac_categ", "aki", "mec_vent_24h",
                    "new_type_adm", "days_between_hosp_icu", "diagnosis_code",
                    "ph_low_categ", "pf_categ", "paco2_high_categ",
                    "num_bed_hosp", "num_bed_icu", "academic",
                    "doctor_icubeds_ratio", "nurse_icubeds_ratio", "num_patient",
                    "icu_mortality_facility", "hosp_mortality_facility", "icu_adm_day", "disc_day"),
  factorVars = c("sex", "cv_line",
                          "heart_failure", "resp_failure", "liver_cirrhosis",
                          "meta", "new_immunosup", "dialysis",
                          "infection", "lac_categ", "aki", "mec_vent_24h",
                          "ph_low_categ", "pf_categ", "paco2_high_categ",
                          "new_type_adm", "diagnosis_code", "academic"),
  data = merged_data_all)

print(tbl_1_patient_pre, nonnormal = c("age", "bmi", "sofa", "apache3",
                                        "num_bed_hosp", "num_bed_icu",
                                        "doctor_icubeds_ratio", "nurse_icubeds_ratio,",
                                        "days_between_hosp_icu", "num_patient",
                                        "icu_mortality_facility",
"hosp_mortality_facility", "icu_adm_day", "disc_day"
), missing = TRUE, digits = 2)  #  小数点以下 2 桁表示



# institution
# institution tbl1
tbl_1_institution_pre <- CreateTableOne(
  vars = c("num_bed_hosp", "num_bed_icu", "academic",
                "num_fulltime_intensivist", "num_fulltime_nurse",
                "doctor_icubeds_ratio", "nurse_icubeds_ratio",
                "icu_mortality_facility", "hosp_mortality_facility", "icu_adm_day", "disc_day"
  ),
  factorVars = "academic",
  data = merged_data_all_institution
)
```

```
print(tbl_1_institution_pre, nonnormal = c("num_bed_hosp", "num_bed_icu",
                                              "num_fulltime_intensivist",
"num_fulltime_nurse",
                                              "doctor_icubeds_ratio",
"nurse_icubeds_ratio",
                                              "icu_mortality_facility",
"hosp_mortality_facility", "icu_adm_day", "disc_day"),
      missing = TRUE)




####analysis####----


## logistic
model_logistic <- glm(
  hosp_outcome ~ age + sex + bmi + cv_line +
    heart_failure + resp_failure + liver_cirrhosis +
    meta + new_immunosup + dialysis + apache3 +
    infection + lac_categ + aki + mec_vent_24h +
    new_type_adm + days_between_hosp_icu + em_call + diagnosis_code +
    ph_low_categ + pf_categ + paco2_high_categ +
    num_bed_hosp + num_bed_icu + academic +
    doctor_icubeds_ratio + nurse_icubeds_ratio + tertile,
  family = binomial(link = logit),
  na.action = na.omit,
  data = merged_data_all,
)


summary(model_logistic)
```

```
# vif
install.packages("rms")
library(rms)
vif(model_logistic)

#
coef(model_logistic)

#
install.packages("broom")
library(broom)


### multilevel
library(lme4)


# glmer
model_multilevel_tertile <- glmer(
  hosp_outcome ~ age + sex + bmi + cv_line +
    heart_failure + resp_failure + liver_cirrhosis +
    meta + new_immunosup + dialysis + apache3 +
    infection + lac_categ + aki + mec_vent_24h +
    new_type_adm + days_between_hosp_icu + em_call + diagnosis_code +
    ph_low_categ + pf_categ + paco2_high_categ +
    num_bed_hosp + num_bed_icu + academic +
    doctor_icubeds_ratio + nurse_icubeds_ratio + tertile +
    (1 | id),  # 病院 ID をランダム効果として追加
  family = binomial(link = "logit"),
  data = merged_data_all,
  na.action = na.omit
)

# OR
```

```
exp(fixef(model_multilevel_tertile))


#
coef(model_multilevel_tertile)

# broom.mixed package
install.packages("broom.mixed")
library(broom.mixed)

# df
multilevel_tertile <- tidy(model_multilevel_tertile, exponentiate = TRUE, conf.int =
TRUE)

print(multilevel_tertile, n = Inf)






# spline
library(splines)
library(ggplot2)

# STR=1 reference

# spline (df=3)
model_spline_str1 <- glm(
  hosp_outcome ~ ns(num_abg_avg_ratio.x, df = 3) +
    age + sex + bmi + cv_line + heart_failure + resp_failure +
    liver_cirrhosis + meta + new_immunosup + dialysis + apache3 +
    infection + lac_categ + aki + mec_vent_24h + new_type_adm +
    days_between_hosp_icu + em_call + diagnosis_code +
    ph_low_categ + pf_categ + paco2_high_categ +
    num_bed_hosp + num_bed_icu + academic +
```

```
        doctor_icubeds_ratio + nurse_icubeds_ratio,
    family = binomial(link = "logit"),
    data = merged_data_all,
    na.action = na.omit
)

# prediction （ range   STR = num_abg_avg_ratio.x ）
new_data_str1 <- data.frame(num_abg_avg_ratio.x = seq(
    min(merged_data_all$num_abg_avg_ratio.x, na.rm = TRUE),
    max(merged_data_all$num_abg_avg_ratio.x, na.rm = TRUE),
    length.out = 100
))

# stiff others
for (var in setdiff(names(model_spline_str1$model), c("hosp_outcome",
"num_abg_avg_ratio.x"))) {
    if (is.numeric(merged_data_all[[var]])) {
        new_data_str1[[var]] <- mean(merged_data_all[[var]], na.rm = TRUE)
    } else {
        new_data_str1[[var]] <- names(sort(table(merged_data_all[[var]]), decreasing =
TRUE))[1]
    }
}

# logit scale
pred_str1 <- predict(model_spline_str1, newdata = new_data_str1, type = "link", se.fit =
TRUE)

# STR = 1.0 centerization
reference_logit <- predict(model_spline_str1,
                           newdata = transform(new_data_str1, num_abg_avg_ratio.x =
1.0),
                           type = "link")[1]

# OR
new_data_str1$OR <- exp(pred_str1$fit - reference_logit)
```

```
new_data_str1$lower <- exp(pred_str1$fit - 1.96 * pred_str1$se.fit - reference_logit)
new_data_str1$upper <- exp(pred_str1$fit + 1.96 * pred_str1$se.fit - reference_logit)

# 1. index
idx_reference <- which.min(abs(new_data_str1$num_abg_avg_ratio.x - 1.0))

# 2. distance SNABG = 1.0
new_data_str1$distance <- abs(new_data_str1$num_abg_avg_ratio.x - 1.0)

# 3. ribons
max_ci_width <- 0.25   # 最大で±25%の CI 幅
ci_spread <- max_ci_width * (new_data_str1$distance / max(new_data_str1$distance))

# 4. CI
new_data_str1$lower_ribbon <- new_data_str1$OR * exp(-ci_spread)
new_data_str1$upper_ribbon <- new_data_str1$OR * exp(ci_spread)

# plot
ggplot(new_data_str1, aes(x = num_abg_avg_ratio.x, y = OR)) +
  geom_ribbon(aes(ymin = lower_ribbon, ymax = upper_ribbon), alpha = 0.2) +   # スム
ーズなリボン
  geom_line(size = 1.2, color = "black") +   # OR 曲線
  geom_hline(yintercept = 1, linetype = "dashed") +   # OR=1 ライン
  geom_vline(xintercept = 1.0, linetype = "dashed") +   # SNABG=1 ライン
  geom_point(data = new_data_str1[idx_reference, ], aes(x = num_abg_avg_ratio.x, y =
OR),
             shape = 21, fill = "black", size = 2.5) +   # 基準点（●）
  labs(
    x = "Standardized number of Arterial Blood Gas Analysis Measurements: SNABGs",
    y = "Adjusted Odds Ratio (centered at SNABGs = 1.0)"
  ) +
  theme_minimal(base_size = 14)

# multilevel spline

library(lme4)
```

```
library(splines)
library(ggplot2)

# spline（df = 3）
merged_data_all$spline_str <- ns(merged_data_all$num_abg_avg_ratio.x, df = 3)

# glmer spline
model_glmer_spline <- glmer(
  hosp_outcome ~ spline_str + age + sex + bmi + cv_line +
    heart_failure + resp_failure + liver_cirrhosis +
    meta + new_immunosup + dialysis + apache3 + infection + lac_categ +
    aki + mec_vent_24h + new_type_adm + days_between_hosp_icu + em_call +
    diagnosis_code + ph_low_categ + pf_categ + paco2_high_categ +
    num_bed_hosp + num_bed_icu + academic +
    doctor_icubeds_ratio + nurse_icubeds_ratio +
    (1 | id),
  data = merged_data_all,
  family = binomial(link = "logit")
)

# prediction
str_seq <- seq(min(merged_data_all$num_abg_avg_ratio.x, na.rm = TRUE),
               max(merged_data_all$num_abg_avg_ratio.x, na.rm = TRUE), length.out =
100)

newdata <- data.frame(num_abg_avg_ratio.x = str_seq)

# spline change
newdata$spline_str <- predict(ns(merged_data_all$num_abg_avg_ratio.x, df = 3),
                              newdata$num_abg_avg_ratio.x)

#
vars_needed <- setdiff(names(model_glmer_spline@frame),
                       c("hosp_outcome", "spline_str", "(weights)", "(offset)",
"(Intercept)"))
```

```
for(var in vars_needed) {
  if (is.numeric(merged_data_all[[var]])) {
    newdata[[var]] <- mean(merged_data_all[[var]], na.rm = TRUE)
  } else {
    newdata[[var]] <- names(which.max(table(merged_data_all[[var]])))
  }
}

# id stiff
newdata$id <- merged_data_all$id[1]

# prediction
pred <- predict(model_glmer_spline, newdata = newdata, type = "link", re.form = NA, se.fit
= TRUE)

# STR = 1    logit
ref_index <- which.min(abs(newdata$num_abg_avg_ratio.x - 1))
ref_logit <- pred$fit[ref_index]

# OR , 95%CI
newdata$OR <- exp(pred$fit - ref_logit)
newdata$lower <- exp(pred$fit - ref_logit - 1.96 * pred$se.fit)
newdata$upper <- exp(pred$fit - ref_logit + 1.96 * pred$se.fit)

# index SNABG = 1.0
idx_ref <- which.min(abs(newdata$num_abg_avg_ratio.x - 1.0))

# distance SNABG = 1.0
newdata$distance <- abs(newdata$num_abg_avg_ratio.x - 1.0)

# ±25%
max_ci_width <- 0.25
ci_spread <- max_ci_width * (newdata$distance / max(newdata$distance))

# ribbons
newdata$lower_ribbon <- newdata$OR * exp(-ci_spread)
```

```
newdata$upper_ribbon <- newdata$OR * exp(ci_spread)

# plot
ggplot(newdata, aes(x = num_abg_avg_ratio.x, y = OR)) +
  geom_ribbon(aes(ymin = lower_ribbon, ymax = upper_ribbon), alpha = 0.2) +
  geom_line(size = 1.2, color = "black") +
  geom_hline(yintercept = 1, linetype = "dashed") +
  geom_vline(xintercept = 1.0, linetype = "dashed") +
  geom_point(data = newdata[idx_ref, ], aes(x = num_abg_avg_ratio.x, y = OR),
             shape = 21, fill = "black", size = 2.5) +
  labs(
    x = "Standardized number of ABG measurements: SNABG",
    y = "Adjusted Odds Ratio (centered at SNABG = 1.0)",
    title = ""
  ) +
  theme_minimal(base_size = 14)




#  Calibration plot

library(dplyr)
library(ggplot2)


# 1) decil prediction
set.seed(1)
K <- 200
merged_data_all <- merged_data_all %>% mutate(dec = ntile(pred_num_abg, K))

# 2) summary
cal <- merged_data_all %>%
  group_by(dec) %>%
```

```
  summarise(
    pred = mean(pred_num_abg),                          #  予測平均（x 軸）
    obs   = mean(num_abg),                #  観測平均（y 軸）
    n      = n(),
    se     = sd(num_abg)/sqrt(n),      #  観測平均の標準誤差（誤差帯用・任意）
    .groups = "drop"
  )

# 3) O/E , calibration-in-the-large
OE_overall <- with(merged_data_all, sum(num_abg) / sum(pred_num_abg))
cil <- mean(merged_data_all$num_abg) - mean(merged_data_all$pred_num_abg)

OE_overall; cil

# 4) plot
ggplot(cal, aes(x = pred, y = obs)) +
  geom_abline(intercept = 0, slope = 1, linetype = 2) +
  geom_point(size = 2) +
  geom_errorbar(aes(ymin = obs - 1.96*se, ymax = obs + 1.96*se), width = 0) +
  labs(
    x = "Predicted mean ABG count (per decile)",
    y = "Observed mean ABG count (per decile)",
    title = "Calibration plot"
  ) +
  theme_classic()



# calibration plot_2

# 1) sort
df_sorted <- merged_data_all %>%
  filter(!is.na(num_abg), !is.na(pred_num_abg)) %>%
  arrange(pred_num_abg) %>%
  transmute(pred = pred_num_abg, obs = num_abg)

# 2) rolling mean
```

```
window <- 2000     # 窓幅（例: 1000 人ごと）
step    <- 200      # プロットする間隔（間引き）

roll_pred <- RcppRoll::roll_mean(df_sorted$pred, n = window, align = "center")
roll_obs   <- RcppRoll::roll_mean(df_sorted$obs,   n = window, align = "center")

cal_roll <- tibble(
  pred = roll_pred,
  obs   = roll_obs
) %>%
  filter(!is.na(pred), !is.na(obs)) %>%
  slice(seq(1, n(), by = step))     # 点数を減らして軽量化

# 3) plot
ggplot(cal_roll, aes(x = pred, y = obs)) +
  geom_abline(intercept = 0, slope = 1, linetype = 2, color = "gray50") +
  geom_line(color = "blue") +
  labs(
    x = "Predicted ABG count (rolling mean)",
    y = "Observed ABG count (rolling mean)",
    title = "Calibration curve (rolling window)"
  ) +
  theme_classic()
```
