# Supplementary material for: Association between ICU-level variation in arterial blood gas utilization and in-hospital mortality: A retrospective cohort study using the Japanese Intensive care PAtient Database registry
Source: PLoS One. 2026 Jun 9;21(6):e0343186. doi: 10.1371/journal.pone.0343186 (PMC13249154; doi:10.1371/journal.pone.0343186)
Supplement: Attachment — Submitted filename: Response to Reviewers.docx [file pone.0343186.s006.docx]

Dear Dr. Emily Chenette

Thank you for your ongoing consideration of our manuscript for publication *PLOS One*. We appreciate the time spent by you and the reviewers and believe the revised manuscript is improved. Below, we have addressed your and the reviewers’ comments.

We look forward to your editorial decision.

Sincerely,

Shunsuke Yawata, MD

Department of Anesthesiology and Critical Care Medicine, Jichi Medical University Saitama Medical Center, Saitama, Japan

Address: 1-847 Amanuma-cho, Omiya Ward, Saitama City, Saitama, Japan

Tel: +81 048-647-2111

E-mail: shunsuke.tottorin@gmail.com

***Additional Editor Comments:***

**Under the title of Outcome Measure and Data Collection, you did not clearly define your main variables and main outcomes. Additionally, you did not provide clear steps for data collection.**

[Response]

We appreciate the editor’s suggestions. We have revised the “Outcome Measures and Data Collection” section to clearly define the primary outcome and exposure, and to improve the description of how data were obtained and variables were defined using the JIPAD registry.

**In your Methods section, you missed mentioning the ethical approval for this study and the permission for data collection.**

[Response]

We thank the editor for the careful review. We have revised the Methods section to explicitly describe the ethical approval, waiver of informed consent, data access period, and that the JIPAD data were accessed and used in accordance with the registry’s data use rules.

**The tables presented are too lengthy.**

[Response]

We have revised Table 1 and Table 3 to improve clarity and readability. Key variables are now presented in the main tables, while detailed categorical variables and the full multivariable model have been moved to the supplementary materials (Tables S2 and S3). We have also added footnotes to the main tables to guide readers to the corresponding supplementary information.

**You have several limitations that should be disclosed to the readers, particularly concerning the nature of the retrospective study and the limitations associated with controlling all confounding variables regarding the direct relationship between the two main variables of this study.**

[Response]

We agree that the limitations inherent to a retrospective observational study, including the potential for residual confounding, should be clearly described. We have revised the Discussion by adding a third limitation in the Strengths and Limitations section. In this addition, we explicitly address unmeasured confounding by providing concrete examples of both patient-level factors (e.g., treatment limitations) and ICU-level factors (e.g., care intensity), and by discussing the potential direction of bias these factors may introduce.

***Review Comments to the Author:***

**Reviewer #1**

**1. JIPAD registry prioritizes recording the "six worst values" if more than six measurements are obtained within 24 hours. As acknowledged in Lines 331–333, this likely underestimates the true frequency of ABGs in high-utilization ICUs. This ceiling effect may obscure the true relationship between very high-intensity monitoring and patient outcomes, potentially biasing the tertile classifications.**

**The authors should discuss how this "ceiling" might have specifically impacted the SNABG calculation and whether a sensitivity analysis excluding patients with exactly six entries was considered to assess this impact.**

[Response]

We appreciate these helpful suggestions. We agree that the truncation of ABG measurements at six values per patient may introduce a ceiling effect, particularly in high-utilization ICUs, which we have described as a limitation. In response to your comment, we have further clarified that this truncation may also distort the distribution of ABG counts, potentially leading to misclassification across tertiles (e.g., patients from the upper range of the middle tertile being classified into the highest tertile). This may reduce the contrast between groups and attenuate the observed association.

We considered a sensitivity analysis excluding patients with six recorded ABG measurements; however, such an approach would restrict the analysis to patients with low to moderate testing frequency and would not resolve the underlying ceiling effect. Therefore, we did not perform this analysis.

**2.The JIPAD registry consists primarily of academic and tertiary centers. As noted in Lines 339–341, these findings may not be generalizable to community or non-tertiary ICUs where staffing levels and physiological monitoring cultures may differ significantly. Staffing ratios (nurse-to-bed) are already quite high in this sample (median 3.30).**

**Provide further detail in the Discussion on how practice patterns might differ in smaller hospitals that lack the 24/7 intensivist presence common in the study's centers.**

[Response]

We agree that practice patterns may differ in smaller or non-tertiary hospitals. However, as our study was based on the JIPAD registry, which primarily includes academic and tertiary centers, it is difficult to directly assess or predict how monitoring practices differ in other settings. We have revised the Discussion to acknowledge that differences in organizational structure and resource availability may influence monitoring strategies, including ABG utilization, and may limit the generalizability of our findings.

**3.The restricted cubic spline analysis suggests a "downward-sloping curve beyond the average value," yet the 95% CI includes 1.0 across most ranges. There is a risk that readers may over-interpret the "tendency toward better outcomes" mentioned in the Conclusion (Line 349) as clinical evidence for higher utilization.**

**The conclusion should more explicitly state that while a trend was visible, it lacked statistical significance at most points on the spline curve to avoid the perception that "more is better" in the acute phase.**

[Response]

We agree that the observed non-linear trend could be over-interpreted as evidence supporting higher ABG utilization. We have revised the Conclusion to explicitly clarify that, although a tendency toward better outcomes at higher utilization levels was observed in the spline analysis, the 95% confidence intervals included 1.0 across most of the range, indicating a lack of statistical significance. We have also emphasized that higher utilization was not consistently associated with benefit.

**4.Most missing values were for BMI (Line 127), leading to the exclusion of 872 patients. This is minor given the sample size but should be noted as a potential source of very small-scale selection bias.**

[Response]

We agree that exclusion of patients with missing BMI values may introduce a small degree of selection bias. In addition, the direction of this bias is unclear, making it difficult to determine its potential impact on the results.

We have revised the manuscript to explicitly acknowledge this limitation while also noting that the proportion of missing data was low (0.7%), and any resulting effect is likely to be minimal.

**Reviewer #2**

**Introduction**

**o The introduction is too long and needs to be summarized.**

**o The first paragraph of the introduction is unnecessary.**

**o Please clarify the study outcomes clearly at the end of the introduction**

[Response]

We thank the reviewer for highlighting these important issues. We have revised the Introduction to improve clarity and focus. Specifically, we removed the original opening paragraph and streamlined the text to more directly present the clinical question. The concept of “less is more” has been retained but moved to the Discussion section to improve the flow of the Introduction. We also clarified the study outcome at the end of the Introduction by explicitly specifying in-hospital mortality.

These revisions have shortened the Introduction and improved the overall structure and readability.

**Results**

**The study flow indicates that 130,994 patients were excluded for the following reasons: age under 16 years while the inclusion criteria involved the patients above 18 years. Please explain.**

[Response]

We thank the reviewer for the careful review. We have carefully reviewed the manuscript. The age criterion used in the study flow was correct, and the corresponding description in the inclusion criteria was incorrect. We have revised the manuscript to clarify that patients younger than 16 years were excluded.

**Discussion**

**The discussion is well-written; however, the clinical significance of the study should be discussed in details.**

[Response]

We agree that the clinical significance of our findings should be more clearly discussed. The Discussion already emphasized that ABG utilization should not be interpreted based on frequency alone and highlighted the importance of context-dependent decision-making in the acute phase of critical illness. We have further strengthened this section by describing the clinical implications of our findings, including that simple strategies based solely on increasing or reducing testing frequency are unlikely to improve outcomes.

**Reviewer #3**

**Rigorous study. Suggest to modify the title make it more understandable. Good discussion yet, ABG should be individualized based on cases rather than the same number of time or less with every patient. Seems like a prevalence study.**

[Response]

We thank the reviewer for the positive and insightful comments. We have revised the title to improve clarity and readability. The updated title now explicitly states the relationship being examined:“Association between ICU-level variation in arterial blood gas utilization and in-hospital mortality.”

We agree that ABG utilization should be individualized based on patient condition rather than applied uniformly across patients. This perspective is consistent with our findings.

We also agree that our study includes an important descriptive aspect of ABG utilization, as suggested by your comment regarding prevalence. We consider ICU-level variation in ABG utilization itself to be a meaningful finding, as illustrated in Figure 2. Building on this, our study further examined the association between ABG utilization and in-hospital mortality. In addition, we have revised the title to improve clarity and readability, as suggested.
