# Supplementary material for: Association between ICU-level variation in arterial blood gas utilization and in-hospital mortality: A retrospective cohort study using the Japanese Intensive care PAtient Database registry
Source: PLoS One. 2026 Jun 9;21(6):e0343186. doi: 10.1371/journal.pone.0343186 (PMC13249154; doi:10.1371/journal.pone.0343186)
Supplement: Peer review history

# Peer review: Decision Letter 0

**DOI:** 10.1371/journal.pone.0343186.r001

**Authors:** Eman Arafa Hassan

13 Apr 2026

PONE-D-26-05297

ICU-level variation in arterial blood gas utilization and patient in-hospital mortality in critically ill patients: A retrospective cohort study using the Japanese Intensive care PAtient Database registry

PLOS One

Dear Dr. Yawata,

Thank you for submitting your manuscript to PLOS ONE. After careful consideration, we feel that it has merit but does not fully meet PLOS ONE’s publication criteria as it currently stands. Therefore, we invite you to submit a revised version of the manuscript that addresses the points raised during the review process.

Please submit your revised manuscript by May 28 2026 11:59PM. If you will need more time than this to complete your revisions, please reply to this message or contact the journal office at plosone@plos.org. When you're ready to submit your revision, log on to https://www.editorialmanager.com/pone/ and select the 'Submissions Needing Revision' folder to locate your manuscript file.

Please include the following items when submitting your revised manuscript:

- A letter that responds to each point raised by the academic editor and reviewer(s). You should upload this letter as a separate file labeled 'Response to Reviewers'.
- A marked-up copy of your manuscript that highlights changes made to the original version. You should upload this as a separate file labeled 'Revised Manuscript with Track Changes'.
- An unmarked version of your revised paper without tracked changes. You should upload this as a separate file labeled 'Manuscript'.

If you would like to make changes to your financial disclosure, please include your updated statement in your cover letter. Guidelines for resubmitting your figure files are available below the reviewer comments at the end of this letter.

If applicable, we recommend that you deposit your laboratory protocols in protocols.io to enhance the reproducibility of your results. Protocols.io assigns your protocol its own identifier (DOI) so that it can be cited independently in the future. For instructions see: https://journals.plos.org/plosone/s/submission-guidelines#loc-laboratory-protocols. Additionally, PLOS ONE offers an option for publishing peer-reviewed Lab Protocol articles, which describe protocols hosted on protocols.io. Read more information on sharing protocols at https://plos.org/protocols?utm_medium=editorial-email&utm_source=authorletters&utm_campaign=protocols.

As the corresponding author, your ORCID iD is verified in the submission system and will appear in the published article. PLOS supports the use of ORCID, and we encourage all coauthors to register for an ORCID iD and use it as well. Please encourage your coauthors to verify their ORCID iD within the submission system before final acceptance, as unverified ORCID iDs will not appear in the published article. *Only* the individual author can complete the verification step; PLOS staff *cannot* verify ORCID iDs on behalf of authors.

We look forward to receiving your revised manuscript.

Kind regards,

Eman Arafa Hassan

Academic Editor

PLOS One

Journal Requirements:

When submitting your revision, we need you to address these additional requirements.

1. Please ensure that your manuscript meets PLOS ONE's style requirements, including those for file naming. The PLOS ONE style templates can be found at

https://journals.plos.org/plosone/s/file?id=wjVg/PLOSOne_formatting_sample_main_body.pdf and

https://journals.plos.org/plosone/s/file?id=ba62/PLOSOne_formatting_sample_title_authors_affiliations.pdf

2. Thank you for stating the following in the Competing Interests section:

"I have read the journal's policy and the authors of this manuscript have the following competing interests: [Shinshu Katayama reports research contracts from Drägerwerk AG & Co. KGaA, Technical University of Munich, Ebenbuild, and Ziosoft, outside the submitted work, and consulting fees from Hamilton Medical AG. All other authors declare no competing interests.]"

Please confirm that this does not alter your adherence to all PLOS ONE policies on sharing data and materials, by including the following statement: "This does not alter our adherence to  PLOS ONE policies on sharing data and materials.” (as detailed online in our guide for authors http://journals.plos.org/plosone/s/competing-interests). If there are restrictions on sharing of data and/or materials, please state these. Please note that we cannot proceed with consideration of your article until this information has been declared.

Please include your updated Competing Interests statement in your cover letter; we will change the online submission form on your behalf.

3. We note that you have indicated that there are restrictions to data sharing for this study. PLOS only allows data to be available upon request if there are legal or ethical restrictions on sharing data publicly. For more information on unacceptable data access restrictions, please see http://journals.plos.org/plosone/s/data-availability#loc-unacceptable-data-access-restrictions.

Before we proceed with your manuscript, please address the following prompts:

a) If there are ethical or legal restrictions on sharing a de-identified data set, please explain them in detail (e.g., data contain potentially identifying or sensitive patient information, data are owned by a third-party organization, etc.) and who has imposed them (e.g., a Research Ethics Committee or Institutional Review Board, etc.). Please also provide contact information for a data access committee, ethics committee, or other institutional body to which data requests may be sent.

b) If there are no restrictions, please upload the minimal anonymized data set necessary to replicate your study findings to a stable, public repository and provide us with the relevant URLs, DOIs, or accession numbers. For a list of recommended repositories, please see

https://journals.plos.org/plosone/s/recommended-repositories. You also have the option of uploading the data as Supporting Information files, but we would recommend depositing data directly to a data repository if possible.

We will update your Data Availability statement on your behalf to reflect the information you provide.

4. If the reviewer comments include a recommendation to cite specific previously published works, please review and evaluate these publications to determine whether they are relevant and should be cited. There is no requirement to cite these works unless the editor has indicated otherwise.

Additional Editor Comments:

Thank you for your submission and for sharing your research with us. I appreciate the effort and dedication you have put into your manuscript.

After careful consideration, I would like to provide some feedback that I believe will enhance the clarity and impact of your work.

Under the title of Outcome Measure and Data Collection, you did not clearly define your main variables and main outcomes. Additionally, you did not provide clear steps for data collection.

In your Methods section, you missed mentioning the ethical approval for this study and the permission for data collection.

The tables presented are too lengthy.

You have several limitations that should be disclosed to the readers, particularly concerning the nature of the retrospective study and the limitations associated with controlling all confounding variables regarding the direct relationship between the two main variables of this study.

[Note: HTML markup is below. Please do not edit.]

Reviewers' comments:

Reviewer's Responses to Questions

**Comments to the Author**

1. Is the manuscript technically sound, and do the data support the conclusions?

The manuscript must describe a technically sound piece of scientific research with data that supports the conclusions. Experiments must have been conducted rigorously, with appropriate controls, replication, and sample sizes. The conclusions must be drawn appropriately based on the data presented.

Reviewer #1: Yes

Reviewer #2: Yes

Reviewer #3: Yes

**********

2. Has the statistical analysis been performed appropriately and rigorously?

Reviewer #1: Yes

Reviewer #2: Yes

Reviewer #3: Yes

**********

3. Have the authors made all data underlying the findings in their manuscript fully available?

The PLOS Data policy requires authors to make all data underlying the findings described in their manuscript fully available without restriction, with rare exception (please refer to the Data Availability Statement in the manuscript PDF file). The data should be provided as part of the manuscript or its supporting information, or deposited to a public repository. For example, in addition to summary statistics, the data points behind means, medians and variance measures should be available. If there are restrictions on publicly sharing data—e.g. participant privacy or use of data from a third party—those must be specified.

Reviewer #1: Yes

Reviewer #2: Yes

Reviewer #3: Yes

**********

4. Is the manuscript presented in an intelligible fashion and written in standard English?

PLOS ONE does not copyedit accepted manuscripts, so the language in submitted articles must be clear, correct, and unambiguous. Any typographical or grammatical errors should be corrected at revision, so please note any specific errors here.

Reviewer #1: Yes

Reviewer #2: Yes

Reviewer #3: Yes

**********

5. Review Comments to the Author

Please use the space provided to explain your answers to the questions above. You may also include additional comments for the author, including concerns about dual publication, research ethics, or publication ethics. (Please upload your review as an attachment if it exceeds 20,000 characters)

Reviewer #1: This retrospective multicenter cohort study examines the association between the frequency of arterial blood gas (ABG) testing within the first 24 hours of ICU admission and in-hospital mortality. Utilizing data from 117,546 patients across 87 Japanese ICUs, the authors standardized ABG utilization to account for patient severity and found wide institutional variation. The study concludes that while early ABG use varies significantly, it is not significantly associated with mortality, though a non-linear trend suggests potential benefits at higher utilization levels. The work is novel as it is the first to directly quantify institutional-level variation in early ABG utilization and its impact on outcomes using a standardized measure.

1. JIPAD registry prioritizes recording the "six worst values" if more than six measurements are obtained within 24 hours. As acknowledged in Lines 331–333, this likely underestimates the true frequency of ABGs in high-utilization ICUs. This ceiling effect may obscure the true relationship between very high-intensity monitoring and patient outcomes, potentially biasing the tertile classifications.

The authors should discuss how this "ceiling" might have specifically impacted the SNABG calculation and whether a sensitivity analysis excluding patients with exactly six entries was considered to assess this impact.

2. The JIPAD registry consists primarily of academic and tertiary centers. As noted in Lines 339–341, these findings may not be generalizable to community or non-tertiary ICUs where staffing levels and physiological monitoring cultures may differ significantly. Staffing ratios (nurse-to-bed) are already quite high in this sample (median 3.30).

Provide further detail in the Discussion on how practice patterns might differ in smaller hospitals that lack the 24/7 intensivist presence common in the study's centers.

3. The restricted cubic spline analysis suggests a "downward-sloping curve beyond the average value," yet the 95% CI includes 1.0 across most ranges. There is a risk that readers may over-interpret the "tendency toward better outcomes" mentioned in the Conclusion (Line 349) as clinical evidence for higher utilization.

The conclusion should more explicitly state that while a trend was visible, it lacked statistical significance at most points on the spline curve to avoid the perception that "more is better" in the acute phase.

4. Most missing values were for BMI (Line 127), leading to the exclusion of 872 patients. This is minor given the sample size but should be noted as a potential source of very small-scale selection bias.

Reviewer #2: Introduction

o The introduction is too long and needs to be summarized.

o The first paragraph of the introduction is unnecessary.

o Please clarify the study outcomes clearly at the end of the introduction

Methods

Methods section is well conducted and well-written

Results

The study flow indicates that 130,994 patients were excluded for the following reasons: age under 16 years while the inclusion criteria involved the patients above 18 years. Please explain.

Discussion

The discussion is well-written; however, the clinical significance of the study should be discussed in details.

Reviewer #3: Rigorous study. Suggest to modify the title make it more understandable. Good discussion yet, ABG should be individualized based on cases rather than the same number of time or less with every patient. Seems like a prevalence study.

**********

6. PLOS authors have the option to publish the peer review history of their article (what does this mean?). If published, this will include your full peer review and any attached files.

If you choose “no”, your identity will remain anonymous but your review may still be made public.

**Do you want your identity to be public for this peer review?** For information about this choice, including consent withdrawal, please see our Privacy Policy.

Reviewer #1: No

Reviewer #2: No

Reviewer #3: No

**********

[NOTE: If reviewer comments were submitted as an attachment file, they will be attached to this email and accessible via the submission site. Please log into your account, locate the manuscript record, and check for the action link "View Attachments". If this link does not appear, there are no attachment files.]

To ensure your figures meet our technical requirements, please review our figure guidelines: https://journals.plos.org/plosone/s/figures

You may also use PLOS’s free figure tool, NAAS, to help you prepare publication quality figures: https://journals.plos.org/plosone/s/figures#loc-tools-for-figure-preparation.

NAAS will assess whether your figures meet our technical requirements by comparing each figure against our figure specifications.

# Author comment: Author response to Decision Letter 1

**DOI:** 10.1371/journal.pone.0343186.r002

28 Apr 2026

Additional Editor Comments:

Under the title of Outcome Measure and Data Collection, you did not clearly define your main variables and main outcomes. Additionally, you did not provide clear steps for data collection.

[Response]

We appreciate the editor’s suggestions. We have revised the “Outcome Measures and Data Collection” section to clearly define the primary outcome and exposure, and to improve the description of how data were obtained and variables were defined using the JIPAD registry.

In your Methods section, you missed mentioning the ethical approval for this study and the permission for data collection.

[Response]

We thank the editor for the careful review. We have revised the Methods section to explicitly describe the ethical approval, waiver of informed consent, data access period, and that the JIPAD data were accessed and used in accordance with the registry’s data use rules.

The tables presented are too lengthy.

[Response]

We have revised Table 1 and Table 3 to improve clarity and readability. Key variables are now presented in the main tables, while detailed categorical variables and the full multivariable model have been moved to the supplementary materials (Tables S2 and S3). We have also added footnotes to the main tables to guide readers to the corresponding supplementary information.

You have several limitations that should be disclosed to the readers, particularly concerning the nature of the retrospective study and the limitations associated with controlling all confounding variables regarding the direct relationship between the two main variables of this study.

[Response]

We agree that the limitations inherent to a retrospective observational study, including the potential for residual confounding, should be clearly described. We have revised the Discussion by adding a third limitation in the Strengths and Limitations section. In this addition, we explicitly address unmeasured confounding by providing concrete examples of both patient-level factors (e.g., treatment limitations) and ICU-level factors (e.g., care intensity), and by discussing the potential direction of bias these factors may introduce.

Review Comments to the Author:

Reviewer #1

1. JIPAD registry prioritizes recording the "six worst values" if more than six measurements are obtained within 24 hours. As acknowledged in Lines 331–333, this likely underestimates the true frequency of ABGs in high-utilization ICUs. This ceiling effect may obscure the true relationship between very high-intensity monitoring and patient outcomes, potentially biasing the tertile classifications.

The authors should discuss how this "ceiling" might have specifically impacted the SNABG calculation and whether a sensitivity analysis excluding patients with exactly six entries was considered to assess this impact.

[Response]

We appreciate these helpful suggestions. We agree that the truncation of ABG measurements at six values per patient may introduce a ceiling effect, particularly in high-utilization ICUs, which we have described as a limitation. In response to your comment, we have further clarified that this truncation may also distort the distribution of ABG counts, potentially leading to misclassification across tertiles (e.g., patients from the upper range of the middle tertile being classified into the highest tertile). This may reduce the contrast between groups and attenuate the observed association.

We considered a sensitivity analysis excluding patients with six recorded ABG measurements; however, such an approach would restrict the analysis to patients with low to moderate testing frequency and would not resolve the underlying ceiling effect. Therefore, we did not perform this analysis.

2.The JIPAD registry consists primarily of academic and tertiary centers. As noted in Lines 339–341, these findings may not be generalizable to community or non-tertiary ICUs where staffing levels and physiological monitoring cultures may differ significantly. Staffing ratios (nurse-to-bed) are already quite high in this sample (median 3.30).

Provide further detail in the Discussion on how practice patterns might differ in smaller hospitals that lack the 24/7 intensivist presence common in the study's centers.

[Response]

We agree that practice patterns may differ in smaller or non-tertiary hospitals. However, as our study was based on the JIPAD registry, which primarily includes academic and tertiary centers, it is difficult to directly assess or predict how monitoring practices differ in other settings. We have revised the Discussion to acknowledge that differences in organizational structure and resource availability may influence monitoring strategies, including ABG utilization, and may limit the generalizability of our findings.

3.The restricted cubic spline analysis suggests a "downward-sloping curve beyond the average value," yet the 95% CI includes 1.0 across most ranges. There is a risk that readers may over-interpret the "tendency toward better outcomes" mentioned in the Conclusion (Line 349) as clinical evidence for higher utilization.

The conclusion should more explicitly state that while a trend was visible, it lacked statistical significance at most points on the spline curve to avoid the perception that "more is better" in the acute phase.

[Response]

We agree that the observed non-linear trend could be over-interpreted as evidence supporting higher ABG utilization. We have revised the Conclusion to explicitly clarify that, although a tendency toward better outcomes at higher utilization levels was observed in the spline analysis, the 95% confidence intervals included 1.0 across most of the range, indicating a lack of statistical significance. We have also emphasized that higher utilization was not consistently associated with benefit.

4.Most missing values were for BMI (Line 127), leading to the exclusion of 872 patients. This is minor given the sample size but should be noted as a potential source of very small-scale selection bias.

[Response]

We agree that exclusion of patients with missing BMI values may introduce a small degree of selection bias. In addition, the direction of this bias is unclear, making it difficult to determine its potential impact on the results.

We have revised the manuscript to explicitly acknowledge this limitation while also noting that the proportion of missing data was low (0.7%), and any resulting effect is likely to be minimal.

Reviewer #2

Introduction

o The introduction is too long and needs to be summarized.

o The first paragraph of the introduction is unnecessary.

o Please clarify the study outcomes clearly at the end of the introduction

[Response]

We thank the reviewer for highlighting these important issues. We have revised the Introduction to improve clarity and focus. Specifically, we removed the original opening paragraph and streamlined the text to more directly present the clinical question. The concept of “less is more” has been retained but moved to the Discussion section to improve the flow of the Introduction. We also clarified the study outcome at the end of the Introduction by explicitly specifying in-hospital mortality.

These revisions have shortened the Introduction and improved the overall structure and readability.

Results

The study flow indicates that 130,994 patients were excluded for the following reasons: age under 16 years while the inclusion criteria involved the patients above 18 years. Please explain.

[Response]

We thank the reviewer for the careful review. We have carefully reviewed the manuscript. The age criterion used in the study flow was correct, and the corresponding description in the inclusion criteria was incorrect. We have revised the manuscript to clarify that patients younger than 16 years were excluded.

Discussion

The discussion is well-written; however, the clinical significance of the study should be discussed in details.

[Response]

We agree that the clinical significance of our findings should be more clearly discussed. The Discussion already emphasized that ABG utilization should not be interpreted based on frequency alone and highlighted the importance of context-dependent decision-making in the acute phase of critical illness. We have further strengthened this section by describing the clinical implications of our findings, including that simple strategies based solely on increasing or reducing testing frequency are unlikely to improve outcomes.

Reviewer #3

Rigorous study. Suggest to modify the title make it more understandable. Good discussion yet, ABG should be individualized based on cases rather than the same number of time or less with every patient. Seems like a prevalence study.

[Response]

We thank the reviewer for the positive and insightful comments. We have revised the title to improve clarity and readability. The updated title now explicitly states the relationship being examined:“Association between ICU-level variation in arterial blood gas utilization and in-hospital mortality.”

We agree that ABG utilization should be individualized based on patient condition rather than applied uniformly across patients. This perspective is consistent with our findings.

We also agree that our study includes an important descriptive aspect of ABG utilization, as suggested by your comment regarding prevalence. We consider ICU-level variation in ABG utilization itself to be a meaningful finding, as illustrated in Figure 2. Building on this, our study further examined the association between ABG utilization and in-hospital mortality. In addition, we have revised the title to improve clarity and readability, as suggested.

Attachment Submitted filename: Response to Reviewers.docx

# Editor report: Decision Letter 1

**DOI:** 10.1371/journal.pone.0343186.r003

**Authors:** Eman Arafa Hassan

3 May 2026

Association between ICU-level variation in arterial blood gas utilization and in-hospital mortality: A retrospective cohort study using the Japanese Intensive care PAtient Database registry

PONE-D-26-05297R1

Dear Dr. Yawata,

We’re pleased to inform you that your manuscript has been judged scientifically suitable for publication and will be formally accepted for publication once it meets all outstanding technical requirements.

Within one week, you’ll receive an e-mail detailing the required amendments. When these have been addressed, you’ll receive a formal acceptance letter and your manuscript will be scheduled for publication.

An invoice will be generated when your article is formally accepted. Please note, if your institution has a publishing partnership with PLOS and your article meets the relevant criteria, all or part of your publication costs will be covered. Please make sure your user information is up-to-date by logging into Editorial Manager at Editorial Manager® and clicking the ‘Update My Information' link at the top of the page. For questions related to billing, please contact billing support.

If your institution or institutions have a press office, please notify them about your upcoming paper to help maximize its impact. If they’ll be preparing press materials, please inform our press team as soon as possible -- no later than 48 hours after receiving the formal acceptance. Your manuscript will remain under strict press embargo until 2 pm Eastern Time on the date of publication. For more information, please contact onepress@plos.org.

Kind regards,

Eman Arafa Hassan

Academic Editor

PLOS One

Additional Editor Comments (optional):

Reviewers' comments:

# Editor report: Acceptance letter

**DOI:** 10.1371/journal.pone.0343186.r004

**Authors:** Eman Arafa Hassan

PONE-D-26-05297R1

PLOS One

Dear Dr. Yawata,

I'm pleased to inform you that your manuscript has been deemed suitable for publication in PLOS One. Congratulations! Your manuscript is now being handed over to our production team.

At this stage, our production department will prepare your paper for publication. This includes ensuring the following:

* All references, tables, and figures are properly cited

* All relevant supporting information is included in the manuscript submission,

* There are no issues that prevent the paper from being properly typeset

You will receive further instructions from the production team, including instructions on how to review your proof when it is ready. Please keep in mind that we are working through a large volume of accepted articles, so please give us a few days to review your paper and let you know the next and final steps.

Lastly, if your institution or institutions have a press office, please let them know about your upcoming paper now to help maximize its impact. If they'll be preparing press materials, please inform our press team within the next 48 hours. Your manuscript will remain under strict press embargo until 2 pm Eastern Time on the date of publication. For more information, please contact onepress@plos.org.

You will receive an invoice from PLOS for your publication fee after your manuscript has reached the completed accept phase. If you receive an email requesting payment before acceptance or for any other service, this may be a phishing scheme. Learn how to identify phishing emails and protect your accounts at https://explore.plos.org/phishing.

If we can help with anything else, please email us at customercare@plos.org.

Thank you for submitting your work to PLOS ONE and supporting open access.

Kind regards,

PLOS ONE Editorial Office Staff

on behalf of

Assistant Professor Eman Arafa Hassan

Academic Editor

PLOS One
